# The Essential UPP Phosphatase Pair BcrC and UppP Connects Cell Wall Homeostasis during Growth and Sporulation with Cell Envelope Stress Response in *Bacillus subtilis*

**DOI:** 10.3389/fmicb.2017.02403

**Published:** 2017-12-05

**Authors:** Jara Radeck, Nina Lautenschläger, Thorsten Mascher

**Affiliations:** Institute of Microbiology, Technische Universität Dresden, Dresden, Germany

**Keywords:** lipid II, bactoprenol, undecaprenyl pyrophosphate, undecaprenol, undecaprenyl phosphate, bacitracin, cell wall biosynthesis

## Abstract

The bacterial cell wall separates the cell from its surrounding and protects it from environmental stressors. Its integrity is maintained by a highly regulated process of cell wall biosynthesis. The membrane-located lipid II cycle provides cell wall building blocks that are assembled inside the cytoplasm to the outside for incorporation. Its carrier molecule, undecaprenyl phosphate (UP), is then recycled by dephosphorylation from undecaprenyl pyrophosphate (UPP). In *Bacillus subtilis*, this indispensable reaction is catalyzed by the UPP phosphatases BcrC and UppP. Here, we study the physiological function of both phosphatases with respect to morphology, cell wall homeostasis and the resulting cell envelope stress response (CESR). We demonstrate that *uppP* and *bcrC* represent a synthetic lethal gene pair, which encodes an essential physiological function. Accordingly, cell growth and morphology were severely impaired during exponential growth if the overall UPP phosphatase level was limiting. UppP, but not BcrC, was crucial for normal sporulation. Expression of *bcrC*, but not *uppP*, was upregulated in the presence of cell envelope stress conditions caused by bacitracin if UPP phosphatase levels were limited. This homeostatic feedback renders BcrC more important during growth than UppP, particularly in defense against cell envelope stress.

## Introduction

The bacterial cell wall is an essential structure that gives the cell its shape and counteracts the turgor pressure. The sacculus is one large macromolecule made up of peptidoglycan that has amazing properties: It is rigid, yet flexible and is constantly expanded and recycled during growth and cell division in a highly regulated manner, both spatially and temporally (Laddomada et al., 2016). Due to its essentiality, it is a prime antibiotic target at virtually any of the numerous steps leading to cell wall assembly.

**FIGURE 1:**
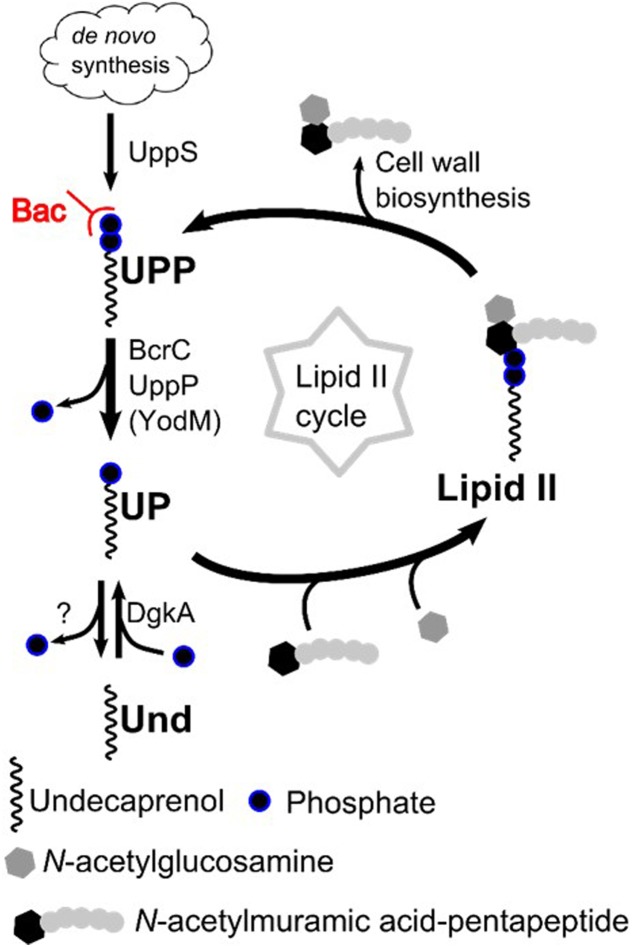
Simplified scheme of the Lipid II-cycle. UPP is dephosphorylated to UP by the UPP phosphatases BcrC, UppP and YodM. The peptide antibiotic bacitracin specifically binds to UPP, thereby inhibiting its dephosphorylation. The carrier UP is loaded with a cell wall precursor, resulting in lipid II. After incorporation of the cell wall precursor into the existing cell wall, UPP is released and recycled by the UPP phosphatases. *De novo* synthesis of UPP occurs from isoprenoids via the enzyme UppS. Undecaprenol can also serve as an unphosphorylated carrier, which is phosphorylated by the kinase DgkA to UP. Bac, Bacitracin; Und, Undecaprenol; UP, Undecaprenyl phosphate; UPP, Undecaprenyl pyrophosphate.

The lipid II cycle describes the membrane-associated steps of this process (**Figure 1**). Briefly, *N*-acetylmuramic acid (MurNAc)-pentapeptide building blocks are assembled in the cytosol and linked to the lipid carrier, a C_55_-phosphate called bactoprenol or undecaprenyl phosphate (UP), thereby forming lipid I. An *N*-acetylglucosamine (GlcNAc) molecule is added, resulting in lipid II. This cell wall building block is subsequently shuttled across the membrane by the flippases Amj and MurJ (Meeske et al., 2015; Laddomada et al., 2016). On the outside, the GlcNac-MurNAc-pentapeptide building block is incorporated into the existing cell wall by transgylcosylation and transpeptidation reactions, thereby releasing the lipid carrier in its pyrophosphate form (undecaprenyl pyrophosphate, UPP). For its recycling, UPP is then dephosphorylated to UP by specialized UPP phosphatases (Bernard et al., 2005; Manat et al., 2014) and flipped back to the cytosolic leaflet of the membrane, where it can be reloaded to enter the Lipid II cycle again.

Apart from this recycling, the cellular UP pool can also be replenished by *de novo* synthesis of UPP via the UPP synthetase UppS (Guo et al., 2005). The subsequent dephosphorylation to UP is likely performed by the same UPP phosphatases that are required for recycling UPP (Manat et al., 2014). In Gram-positive bacteria, UP can also originate from phosphorylating undecaprenol, e.g., by the kinase DgkA in *Bacillus subtilis* (Higashi et al., 1970; Jerga et al., 2007).

UP is the carrier for both peptidoglycan and wall teichoic acids (WTA) building blocks and its availability represents the central bottleneck for the synthesis of lipid II both *in vitro* and *in vivo* (Breukink et al., 2003; Breukink and de Kruijff, 2006; Egan et al., 2015). Only ∼ 2^∗^10^5^ UP molecules (0.5–1% of all phospholipids) are present per cell (Kramer et al., 2004) and it has been estimated that each of the carriers shuttles one to three cell wall building blocks per seconds during exponential growth (McCloskey and Troy, 1980). The amount of WTA and peptidoglycan synthesis is reduced under UP-limitation, especially if conditions favor the competing pathway (Anderson et al., 1972). Antibiotics that target the lipid II cycle benefit from this bottleneck, because blocking any step will lead to accumulation of intermediates, shortage of free carrier molecules and impaired cell wall biosynthesis that depends on UP.

Maintaining envelope integrity is absolutely essential for the survival of any bacterial cell, as are the metabolic processes that ensure it. Bacteria have therefore evolved appropriate countermeasures to detect and remove threats or damages to cell envelope homeostasis before they become lethal. These responses are collectively termed cell envelope stress response (CESR) (Jordan et al., 2008). *Bacillus subtilis* is one of the main model organisms for studying the Gram-positive cell wall and member of the *Firmicutes* phylum (low G + C Gram-positives). In this organism, the CESR is orchestrated by two-component systems and extracytoplasmic sigma factors (ECFs) (Radeck et al., 2016a). While many antibiotics can trigger the CESR, the molecular nature behind these stimuli has only been identified for very few cases. The antibiotic itself seems rarely to be detected directly. Instead, downstream effects of antibiotic threat, such as envelope damage or – more importantly – the accumulation of certain intermediates, are suspected to be the actual triggers of CESR (Meeske et al., 2015; Helmann, 2016). A similar effect to such an antibiotic-mediated blockade can also be achieved by reducing the availability of the corresponding enzyme. Consequently, the lipid II cycle, cell wall homeostasis and cell envelope stress (CES) are interconnected processes that can hardly be studied independently. In fact, a *B. subtilis* mutant with reduced UppS activity (and therefore reduced *de novo* synthesis of UPP) had altered antibiotic resistance properties and elevated σ^M^-activity (Lee and Helmann, 2013). Here, we will focus on the CESR caused by limitations of the crucial UPP phosphatase activity, provided, e.g., by BcrC.

The expression of *bcrC* is controlled by multiple stress-inducible alternative sigma factors, including σ^M^, σ^I^, σ^X^, σ^V^, and potentially also σ^W^ (Cao and Helmann, 2002; Tseng and Shaw, 2008; Guariglia-Oropeza and Helmann, 2011; Zweers et al., 2012). σ^M^ controls approximately 60 genes involved in cell wall synthesis, shape determination, detoxification and DNA damage response (Eiamphungporn and Helmann, 2008). It is activated by multiple triggers, including antibiotics, high salt, heat stress, and acidic pH (Thackray and Moir, 2003). While all of these inducers affect cell envelope synthesis or integrity, the molecular cue for the activation of this and other ECFs is yet to be identified (as reviewed in Helmann, 2016).

Induction of P*_bcrC_* can be triggered, e.g., by the addition of the antibiotic bacitracin (Cao and Helmann, 2002; Radeck et al., 2016b). Bacitracin is a cyclic antimicrobial peptide produced by some strains of *Bacillus licheniformis* and *B. subtilis* (Azevedo et al., 1993; Ishihara et al., 2002). It was shown that bacitracin tightly binds UPP, thereby blocking the dephosphorylation reaction mediated by UPP phosphatases and consequently slowing down the lipid II cycle (Siewert and Strominger, 1967; Storm and Strominger, 1973; Economou et al., 2013). The deletion of *bcrC* may have similar consequences, since the loss of one UPP phosphatase might reduce the rate of UPP dephosphorylation to UP.

A very sensitive indicator of CES is the LiaR-controlled *liaI* promoter (P*_liaI_*) (Mascher et al., 2004). The cognate three-component system, LiaFSR reacts to a broad range of cell envelope stressors, including alkaline shock, oxidative stress, or bacitracin addition (Jordan et al., 2006; Wolf et al., 2010). In turn, it regulates a phage-shock protein-like response that provides a secondary layer of protection against CES (Radeck et al., 2016b). The low basal activity and strong, highly dynamic induction of P*_liaI_* made this promoter an ideal candidate for the development of a highly sensitive CESR-inducible whole cell biosensor (Mascher et al., 2004; Wolf and Mascher, 2016; Kobras et al., 2017). Recently, we demonstrated that P*_liaI_* activity in response to bacitracin is elevated in a *bcrC* deletion mutant and decreased in a *bcrC* overexpression strain. These findings indicate that the CES caused by bacitracin is relieved in the presence of the UPP phosphatase BcrC (Radeck et al., 2016b).

In the same study, we observed that P*_bcrC_* activities were increased in a *bcrC* null mutant. Together, this lead to the hypothesis that changes in UP and UPP levels can be sufficient to create CES (Radeck et al., 2016b). Due to their crucial role in the lipid II cycle, we hypothesize that impaired UPP phosphatase activity leads to a limitation in cell wall synthesis, which in turn should increase the CESR. Here, we aimed at challenging this hypothesis by studying the effects of enzymes potentially involved in UP turnover on *B. subtilis* physiology and stress responses in detail.

The genome of *B. subtilis* encodes three UPP phosphatases, BcrC, UppP and YodM. YodM and BcrC belong to the large group of type II phosphatidic acid phosphatases (PAP2s) that share their catalytic mechanism while pursuing a wide range of functions from signaling to export. Both proteins are homologues to the crystalized UPP phosphatase PgpB of *Escherichia coli* (El Ghachi et al., 2005; Fan et al., 2014; Kelley et al., 2015). While YodM seems to be dysfunctional due to insufficient expression (Zhao et al., 2016), BcrC has been studied to some extent. It seems to be the major *B. subtilis* UPP phosphatase (Bernard et al., 2005; Inaoka and Ochi, 2012) and is highly expressed at most culture conditions, as judged by a comprehensive tiling array study (Supplementary Figure S1) (Nicolas et al., 2012). The monocistronic gene *bcrC* (Supplementary Figure S1) is regulated by the CES-inducible ECFs σ^M^ and σ^X^ (Cao and Helmann, 2002). The latter responds to CES that might be caused by changing UP levels or other intermediates of the lipid II cycle (Helmann, 2016).

The minor UPP phosphatase UppP (Inaoka and Ochi, 2012) is homologous to BacA from *E. coli*. The latter accounts for about 75% of the UPP phosphatase activity in this organism (El Ghachi et al., 2004). *uppP* is the second gene of the *yubA-uppP* operon and its P*_yubA_*-dependent expression is not induced by bacitracin (Cao and Helmann, 2002). YubA is predicted to be a membrane protein belonging to the autoinducer-2 exporter (ai-2e) family and might be associated with cell wall synthesis (Fenton et al., 2016; The UniProt Consortium, 2017).

To investigate the correlation between UPP phosphatases and cell envelope homeostasis, we analyzed strains depleted for (combinations of) both the UPP phosphatases BcrC/UppP and the undecaprenol kinase DgkA on cell physiology and morphology. First, we demonstrate the synthetic lethality of BcrC and UppP and a severe morphological defect in UPP phosphatase depleted strains. Next, UppP is shown to be indispensable for efficient sporulation. Unexpectedly, *uppP* or *bcrC* deletion and complementation mutants did not activate a classical CESR, as judged by the lack of P*_liaI_* induction. Instead, the resulting limitation in UPP phosphatase levels is perceived by the broader ECF-dependent signaling network. As a result, P*_bcrC_* activity was increased in those mutants, thereby providing a homeostatic feedback mechanism by which the cell can autoregulate its UPP phosphatase level according to needs. Furthermore, we provide the first evidence that DgkA is indeed involved in UPP homeostasis: While a lack of this predicted undecaprenol kinase did not result in an observable deficiency, a (most likely minor) role in UP turnover is indicated by an increased activity of P*_bcrC_* in a *dgkA* mutant in stationary phase. Taken together, our data provides the first insight into the fine-tuning of UP homeostasis that adjusts the Lipid II cycle, and hence cell wall biosynthesis, in response to growth rates and envelope stress levels.

## Results

### High Level Expression of the UPP Phosphatase Encoding Genes *bcrC* and *uppP* in *B. subtilis*

**FIGURE 2:**
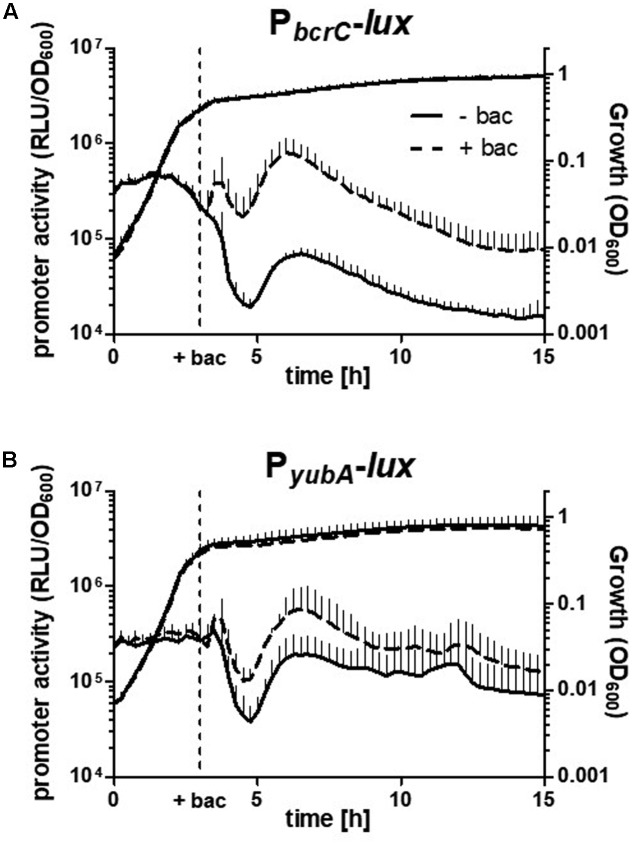
Expression of UPP phosphatase genes. Growth (OD_600_) and activity levels (RLU/OD_600_) of P*_bcrC_* **(A)** and P*_yubA_* **(B)** in *B. subtilis* W168 (TMB1620 and TMB3688) from early exponential to late stationary phase in absence or presence of 30 μg ml^-1^ bacitracin (+ bac; dashed lines), respectively. P*_bcrC_* activity was significantly increased upon bacitracin addition (p = 0.021, 2-way ANOVA), but not P*_yubA_* (*p* = 0.29, 2-way ANOVA). Measurements were obtained in a microtiter plate reader at 37°C in MCSEC medium. Data is shown for three independent biological replicates (mean and SD).

We first wanted to analyze the expression of the two UPP phosphatase-encoding genes *bcrC* and *uppP* by monitoring the activity of strains harboring the corresponding promoter-*lux* fusions, P*_bcrC_* and P*_yubA_*, respectively. Under our experimental conditions, the activity of P*_bcrC_* remained at high levels (**Figure 2A**) from early exponential to late stationary phase - with exception of the known decrease during transition state, which is frequently observed for online promoter activity measurements (Radeck et al., 2013). As discovered previously, P*_bcrC_* activity was increased by the addition of bacitracin (30 μg ml^-1^; **Figure 2A**) (Mascher et al., 2003; Radeck et al., 2016b).

The activity of P*_yubA_* is comparable to P*_bcrC_* during exponential growth, but about three-fold higher during stationary phase (**Figure 2B**). In contrast to P*_bcrC_*, and in agreement with a previous study, P*_yubA_* was not significantly induced by bacitracin (**Figure 2B**, Cao and Helmann, 2002). Our data based on the promoter-*lux* fusions agrees well with the tiling array data on mRNA levels of *uppP* and *bcrC* (Supplementary Figure S1; Nicolas et al., 2012). Under most conditions, *bcrC* is expressed at a slightly higher level than *uppP* (*yubB*), with the exception of sporulation, during which *bcrC* expression drops at early sporulation and *uppP* only at late sporulation (Supplementary Figure S1).

For the third UPP phosphatase, the tiling array data shows that there is almost no transcription of *yodM*, but instead high levels of counter-transcription (Supplementary Figure S1). This finding has recently been verified (Zhao et al., 2016). Due to these observations, YodM and its promoter, P*_yodM_*, were not considered further for our analysis.

Hence, there are two well-transcribed UPP phosphatase genes in *B. subtilis* cells, *bcrC* and *uppP*. We therefore decided to study the role of their gene products in cell wall homeostasis and CES in *B. subtilis*. Toward that goal, we investigated single and combined deletion and complementation strains for their effect on cell morphology, sporulation, CESR and antibiotic resistance.

### *uppP* and *bcrC* Are a Synthetic Lethal Gene Pair

Initially, we aimed at replacing all three UPP phosphatase genes with resistance cassettes (*bcrC*::*tet*, *uppP*::*MLS* and *yodM*::*spc*) in single and double mutants. For simplicity reasons, all allelic replacements are noted as deletions throughout the manuscript and figure legends. All single mutants and double mutants with Δ*yodM* were readily obtained. Since the lack of any observable phenotype during the initial characterization of all Δ*yodM* strains can readily be explained by the lack of *yodM* expression (**Figure 2A** and Zhao et al., 2016), these strains were not considered further.

In contrast to the single mutants, multiple attempts to construct a Δ*uppP ΔbcrC* double mutant failed, indicative of synthetic lethality of *bcrC* and *uppP.* To support this assumption, we constructed complementation strains, in which *uppP* or *bcrC* were ectopically integrated into the *thrC* locus under control of the xylose-inducible promoter P*_xylA_*. In strains carrying a complementation copy of either *bcrC* or *uppP*, the deletion of both native genes was possible in the presence of xylose. These strains (Δ*uppP* Δ*bcrC* P*_xylA_*-*bcrC* and Δ*uppP* Δ*bcrC* P*_xylA_*-*uppP*) will be referred to as depletion strains to distinguish them from the complementation strains Δ*bcrC* P*_xylA_*-*bcrC* and Δ*uppP* P*_xylA_*-*uppP*. Our findings are in agreement with a recent study from the Helmann laboratory, which independently demonstrated the synthetic lethality of the *bcrC*/*uppP* gene pair using a CRISPR-dCas9 knockdown approach (Zhao et al., 2016).

### Cell Morphology Is Impaired in UPP Phosphatase Mutants during Exponential Growth

Depletion of essential envelope-associated proteins often leads to bulging, filamentation or lysis of cells (Peters et al., 2016). Since *uppP* and *bcrC* are synthetic lethal and the lipid II-cycle and cell wall synthesis depend on the recycling of UP by UPP phosphatases, we hypothesized that a depletion of UPP phosphatases in fast-growing cells leads to a morphological phenotype similar to that observed for other essential cell envelope functions.

**FIGURE 3:**
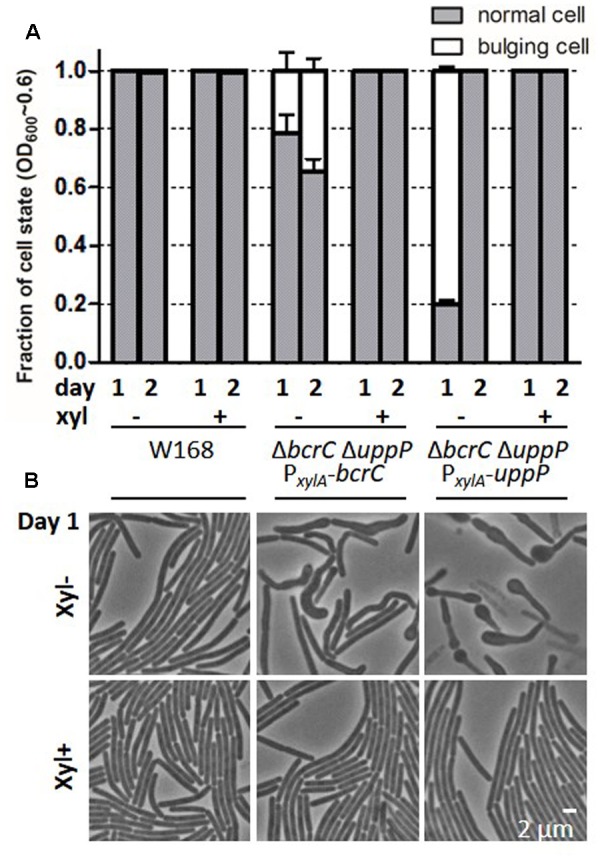
Cell morphology during exponential growth in *bcrC* and *uppP* complementation mutants. Strains W168, TMB3739, TMB3740 were inoculated from fresh overnight cultures (day 1) or 24 h-cultures (on day 2), grown in MCSEC at 37°C, 220 rpm without xylose (xyl-) to deplete the respective UPP phosphatase or with 0.2% xylose (xyl+) to fully induce the production in complementation mutants. Overnight cultures were always supplemented with xylose, whereas the inoculum for day 2 was taken from samples either with (xyl+) or without (xyl-) xylose added. Phase contrast pictures were taken in late exponential phase (OD_600_∼0.6, ∼6 h post-inoculation). **(A)** Fraction of cells with normal (gray) or bulging morphology (white). At least 1000 cells were counted for each of the three independent biological replicates. **(B)** Representative pictures of cells with normal or bulging morphology. Samples were taken from day 1. The scale bar represents 2 μm.

Single *uppP* or *bcrC* deletions, the respective complementation mutants, and the wild type showed no or less than 0.1% misshaped cells (data not shown). In contrast, both UPP phosphatase depletion strains TMB3739 (Δ*uppP* Δ*bcrC* P*_xylA_*-*bcrC*) and TMB3740 (Δ*uppP* Δ*bcrC* P*_xylA_*-*uppP*) showed a severe phenotype during exponential growth phase (**Figure 3**). In the absence of xylose, about 20–30% (TMB3739) or 80% (TMB3740) of the cells were bulging and sometimes bending (**Figure 3A**). This phenotype could be completely suppressed by the addition of xylose, resulting in high expression levels of the complemented UPP phosphatase (**Figure 3B**). This phenotype could not be observed at slow growth, e.g., in stationary phase (Supplementary Figure S2) even though some cells look swollen compared to wild type cells (e.g., the Δ*uppP* mutant TMB3408).

In summary, we could show that a very low expression of only *uppP* or *bcrC* leads to severe morphological changes, e.g., bulging cells during exponential growth – concomitant with depleted peptidoglycan or WTA synthesis (Muchova et al., 2011; Botella et al., 2014) caused by a lack of UP. This phenotype was most severe for the *uppP* depletion strain.

### A *uppP* Mutant Is Impaired in Efficient Sporulation

**FIGURE 4:**
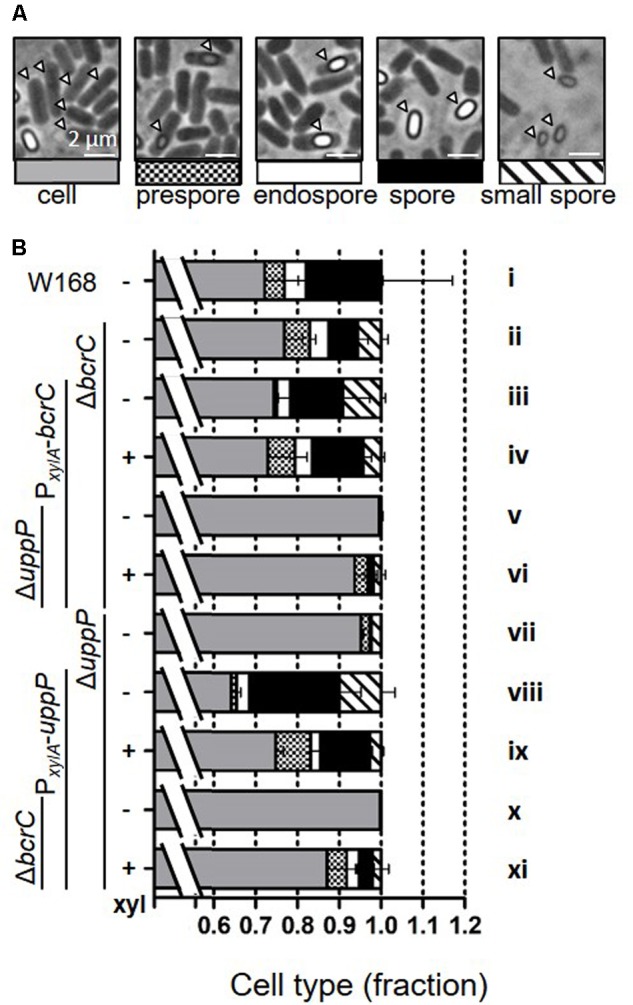
Sporulation efficiencies of *bcrC* and *uppP* deletion and complementation mutants. Strains (W168, TMB0297, TMB3694, TMB3739, TMB3408, TMB3695, and TMB3740) were grown as described in **Figure 3** and phase contrast microscopy pictures were taken 24 h post-inoculation. **(A)** Representative pictures for normal cells (gray), prespores without fully established phase-bright endospore (small checkered), completed endospores (white), free spores (black) and small free spores (striped). **(B)** Cell type fractions are shown as stacked bar graphs. Data is shown for at least 1000 cells per measurement and the error bars represent the standard deviation between independent biological triplicates. The full data set is shown in Supplementary Figure S3.

During the morphology studies, we observed altered sporulation rates between the wild type and UPP phosphatase mutants, especially in Δ*uppP*. We therefore quantified the sporulation efficiency in our strains by determining the fractions of vegetative versus sporulating cells and endospores in a culture 24 h after inoculation (summarized in **Figure 4**, see Supplementary Figure S3 for the complete dataset). Under our experimental conditions, about 30% of the wild type cells (**Figure 4B**, i) were in the process of sporulation or had already sporulated. Mutants with a native copy of *uppP* (ii–iv), and mutants with wild type copy of *bcrC* in combination with an ectopic inducible copy of *uppP* (viii, ix) had similar sporulation rates. Sporulation was impaired (<7%), if the native copy of *uppP* was lost and no ectopic copy was introduced (v, vi), or if *uppP* was depleted in the phosphatase double mutant (x). The sporulation deficiency of the latter could partially be restored by the addition of xylose to induce *uppP* expression (xi).

The reduced sporulation frequencies in Δ*uppP* mutants did not originate from delayed sporulation, since a similar reduction in sporulation rates (2–5% compared to >30% in the wild type) was also observed after 48 h (data not shown). However, using a spore-crust marker (GFP-CotZ), we detected that some of the phase-gray particles in a Δ*uppP* mutant were spores instead of cells (Supplementary Figure S4). This phenotype is indicative of alterations in stage IV or V of sporulation, where mutants have thinner or no germ cell wall or cortex (Coote, 1972; Piggot and Coote, 1976). Both spore layers consist of peptidoglycan, a defect in their synthesis therefore points toward UppP being the responsible UPP phosphatase for the lipid II cycle during sporulation. This observation is in agreement with a recent screen for sporulation mutants, in which a reduced sporulation efficiency and phase-gray spores were also detected in a *uppP* mutant (Meeske et al., 2016).

Here we could show that the rates of normal, phase bright spores drastically decreased in absence of UppP. The physiological relevance of this UPP phosphatase for efficient sporulation is underscored by the dramatic loss in the number of heat resistant spores, which was determined to be only 0,04% of the wild type (Meeske et al., 2016). In the presence of BcrC, low levels of UppP still allow a normal sporulation (**Figure 4**, vii, viii), while this residual UppP amount is not sufficient in the absence of BcrC (x). Either the native copy of *uppP* or the combination of native *bcrC* and an ectopic version of *uppP* is required for efficient sporulation.

The combined results from our sporulation counts (**Figure 4**) and the cell morphology study (**Figure 3**) indicate that limited amounts of either UPP phosphatase alone (TMB3739, TMB3740 without xylose) are not sufficient to retain normal cell shape during fast growth or ensure efficient sporulation. While each native phosphatase is sufficient to keep normal cell shape in exponential growth, BcrC cannot compensate for the lack of UppP during sporulation. Both phenotypes point toward defects in cell wall synthesis. This provoked the question if under such circumstances this bottleneck in cell wall synthesis leads to a CESR, which is normally triggered by the external addition of cell wall antibiotics, such as bacitracin (Radeck et al., 2016a). To address this question, two well established reporters for CESR [the P*_liaI_* and P*_bcrC_* promoters fused to the *lux* reporter cassette (Radeck et al., 2016b)] were combined with the mutant collection and probed for their activity under UPP phosphatase-limiting conditions.

### Limitations in UPP Phosphatases Are Perceived As Envelope Stress by the P*_bcrC_* Reporter

P*_liaI_* is a very sensitive reporter of cell envelope damage due to its wide inducer spectrum and high dynamic activity range (Mascher et al., 2004; Rietkötter et al., 2008). But we did not observe any UPP phosphatase-dependent induction of the P*_liaI_*-controlled CESR, even if we additionally challenged the cells with bacitracin (data not shown).

**FIGURE 5:**
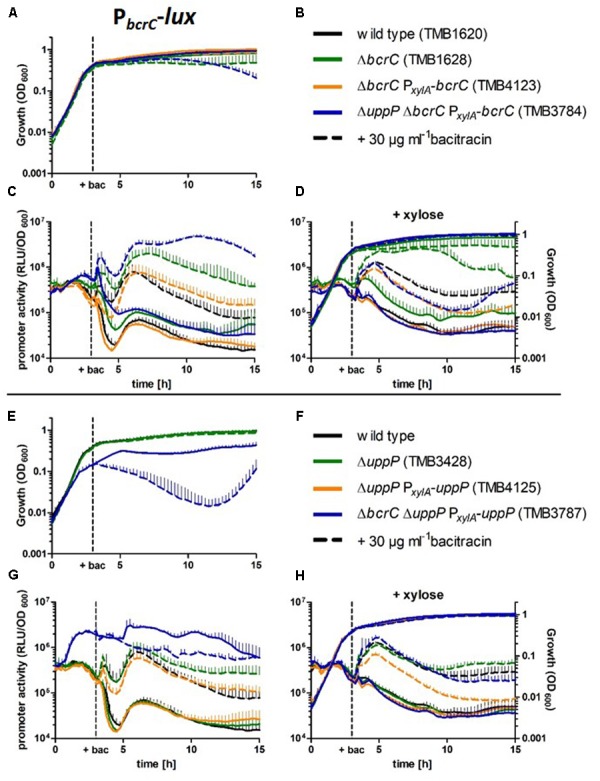
Growth and P*_bcrC_* promoter activities in the wild type and *bcrC* and *uppP* complementation mutants. Strains were grown in MCSEC at 37°C in 96-well plates in a microtiter plate reader. OD_600_ and luminescence was measured every 15 min for 15 h. **(A–D)** Growth and P*_bcrC_*-activity in *bcrC* deletion, complementation and depletion strains. **(E–H)** Growth and P*_bcrC_*-activity in *uppP* deletion, complementation and depletion strains. The strains are defined by the color, while solid or dashed lines indicate the absence or presence of 30 μg ml^-1^ bacitracin. **(D,H)** Samples were grown with 0.2% xylose to fully induce P*_xylA_*-driven gene expression. In these cultures, promoter activity steadily decreased from the transition phase onward. This phenomenon was observed for all strains either harboring P*_bcrC_* or the constitutive reference promoter P*_lepA_* (data not shown). We therefore postulate that the change in promoter dynamics is caused by the presence of an additional C-source (xylose). Thin lines represent the standard deviation of at least three biological replicates.

In contrast to the damage-sensing P*_liaI_* reporter, the P*_bcrC_*-derived reporter is postulated to respond to alterations/limitations in cell wall homeostasis (Minnig et al., 2003) and could therefore be more suitable to detect stress caused by changes in the UPP phosphatase levels. In light of this study, P*_bcrC_* is particularly relevant since it controls the expression of one of the two UPP phosphatases, BcrC. It therefore provides a direct read-out for the cells ability to respond to limitations in UPP phosphatases by upregulating *bcrC* expression. Toward this end, we measured P*_bcrC_*-activity in the wild type as well as *bcrC* and *uppP* deletion, complementation and depletion strains. Promotor activity as relative luminescence units normalized to cell density (RLU/OD_600_) and growth (OD_600_) were measured in a microtiter plate reader for 15 h (**Figure 5**).

For the wild type reporter strain (TMB1620), P*_bcrC_* activity of 3–5^∗^10^5^ RLU/OD_600_ was observed during exponential growth and the transition phase (**Figures 5A,C**; black lines, 0–3 h). The activity decreased about 10-fold during early stationary phase (4–6 h), briefly increased (6–8 h) and then steadily declined during late stationary phase. Upon bacitracin addition (30 μg ml^-1^), the promotor activity was increased about 10-fold, while no change in growth behavior was detected.

In the Δ*bcrC* mutant (TMB1628, green) and the *bcrC* depletion strain (TMB3784, blue), the P*_bcrC_* activity increased without and especially with bacitracin addition and a slightly reduced optical density was observed during stationary phase compared to the wild type (**Figures 5A–D**). These effects were revoked by the addition of xylose (TMB3784) or the introduction of a complementing copy of *bcrC* (TMB4123, orange), even without xylose.

Deletion of *uppP* (TMB3428, green) only had a minor effect on P*_bcrC_* activity (approximately three-fold elevation during late stationary phase upon bacitracin addition, **Figures 5E,G**). However, in the *uppP* depletion strain (TMB3787, blue) impaired growth – especially in the presence of bacitracin – and strongly increased P*_bcrC_* activity was observed throughout growth, even without bacitracin addition. A subsequent in-depth analysis supported these findings: The *uppP* depletion strain (TMB3740), but not the *bcrC* depletion strain (TMB3739) showed a clear growth defect in absence of xylose. All phenotypes of complementation mutants reverted to wild type levels in presence of xylose (see Supplementary Figure S5 for details).

Taken together, the single *bcrC* deletion, as well as two phosphatase depletion strains (Δ*bcrC* Δ*uppP* P*_xylA_*-*bcrC* and Δ*bcrC* Δ*uppP* P*_xylA_*-*uppP*) had the strongest effect on P*_bcrC_* activity, especially in the presence of bacitracin.

### The Undecaprenol Kinase DgkA Contributes to the Cellular UP Pool

The results described in the previous section demonstrate that the cell is indeed capable of perceiving limitations in UPP phosphatase levels, most likely at the level of the resulting UP shortage. A second enzymatic activity potentially contributing to the cellular UP pool is the undecaprenol kinase DgkA that phosphorylates undecaprenol to UP (Jerga et al., 2007). Based on the results of the previous section, the activity of the P*_bcrC_* reporter might provide an ideal read-out to probe if DgkA indeed provides a measurable contribution to the UP pool, particularly if the cellular amount of UPP phosphatases is severely limited. We therefore deleted *dgkA* in the wild type and all phosphatase deletion, complementation and depletion strains and then measured the P*_bcrC_* activity throughout the growth cycle.

**FIGURE 6:**
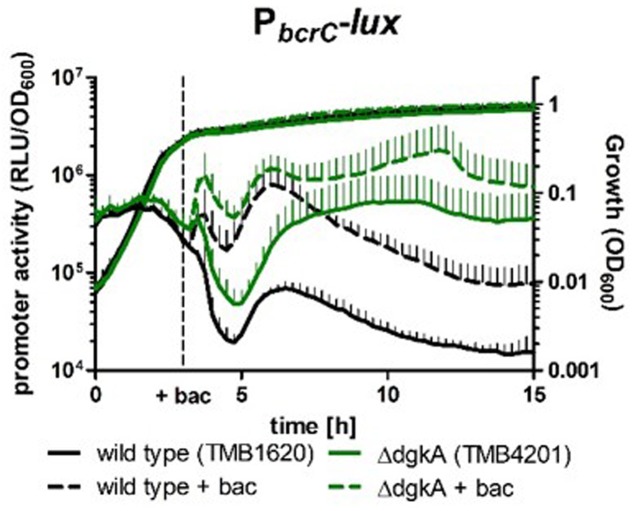
P*_bcrC_* promoter activity depends on DgkA. Strains were grown as described in **Figure 5**. Black, wild type; green, *dgkA* mutant. Samples induced with bacitracin are shown as dashed lines. Thin lines represent the standard deviation of three biological replicates.

Surprisingly, a strong DgkA-dependent effect was already observed in the wild type reporter strain: the P*_bcrC_* activity was elevated ∼10-fold in the *dgkA* mutant during late stationary phase relative to the wild type, both in the presence or absence of bacitracin (**Figure 6**). A similar effect was observed for all phosphatase mutants (Supplementary Figure S6). This result indicates that a DgkA-dependent phosphorylation of undecaprenol indeed detectably contributes to the cellular UP pool, even though a *dgkA* mutant did not show any (additional) morphological phenotype during fast growth (data not shown). It has previously been shown that a *B. subtilis dgkA* mutant produces less and cortex-deficient endospores – a peptidoglycan structure that depends on UP for its synthesis (Amiteye et al., 2003; Supplementary Figure S3). This suggests that the role of DgkA to contribute to the UP pool is rather during sporulation.

### Deletion and Depletion of UPP Phosphatases Increases Sensitivity toward the UPP-Binding Antibiotic Bacitracin

**FIGURE 7:**
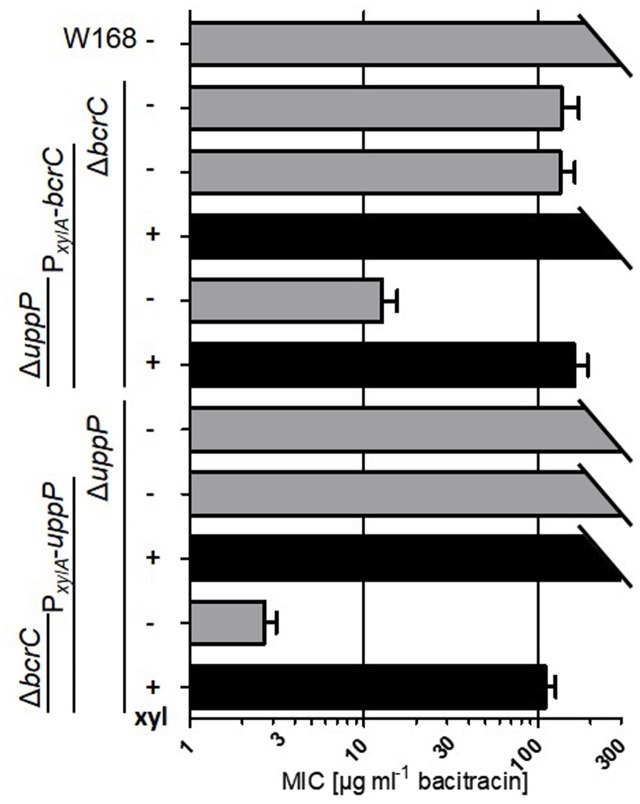
Minimal inhibitory bacitracin concentration of *bcrC* and *uppP* deletion and complementation mutants. Strains (W168, TMB297, TMB3694, TMB3739, TMB3408, TMB3695, and TMB3740) were inoculated from fresh overnight cultures (xyl+) in MCSEC at 37°C with or without 0.2% xylose. During exponential growth, cells were embedded in soft agar and plated as an overlay on MCSEC agar. One Etest^®^ strip (bacitracin 0.016–256 μg ml^-1^) was placed on the soft agar (see Material and Methods). The MIC was determined after 24 h of incubation at 37°C. Data is shown for at least three independent biological replicates (two replicates, if MIC > 256 μg ml^-1^) with mean and standard deviation. The full data set is depicted in Supplementary Figure S7.

*Bacillus subtilis* wild type cells are highly resistant against the UPP-binding antibiotic bacitracin (minimal inhibitory concentration, MIC, >256 μg ml^-1^). The primary resistance determinant is the bacitracin-specific ABC-transporter BceAB (Mascher et al., 2003; Ohki et al., 2003; Rietkötter et al., 2008). But BcrC provides a (secondary) layer of bacitracin resistance, most likely by competing with the antibiotic for the same target molecule, UPP (Bernard et al., 2005; Radeck et al., 2016a,b). The inhibitory effect of bacitracin is based on depleting the UP pool by formation of a UPP-bacitracin complex, finally leading to an arrest of the lipid II cycle (**Figure 1**). It stands to reason to postulate that deletions in genes encoding UPP phosphatases or undecaprenol kinases might also contribute to the sensitivity of the cells toward bacitracin. We therefore measured the MIC for bacitracin in UPP phosphatase deletion and depletion mutants, using Etest^®^ strips (**Figure 7** and Supplementary Figure S7).

While the individual deletion of *uppP* had no measurable effect on bacitracin MIC, the deletion of *bcrC* lead to the known reduction of the MIC to ∼ 120 μg ml^-1^. This phenotype could be complemented by the addition of xylose, thereby inducing the ectopically integrated P*_xylA_*-*bcrC*. Without xylose, the MIC of this strain is comparable to the *bcrC* deletion mutant, indicating very little background activity of P*_xylA_* under non-inducing conditions. If *uppP* is deleted in this genetic background, the MIC was even further decreased to ∼15 μg ml^-1^. In this depletion strain, the P*_xylA_*-mediated expression of *bcrC* can no longer fully compensate for the loss of both UPP phosphatases (MIC of ∼150 compared to >256 μg ml^-1^ in Δ*uppP*). The *uppP* depletion mutant (TMB3739, Δ*bcrC* Δ*uppP* P*_xylA_*-*uppP*) exhibited the lowest MIC (∼3 μg ml^-1^). Upon addition of xylose, a 35-fold increase in bacitracin MIC could be observed (**Figure 7**).

Taken together, the resistance toward the UPP-binding bacitracin is indeed severely reduced in mutants limited for UPP phosphatases. This phenotype can be (almost fully) compensated for by induction of ectopically integrated UPP phosphatase genes under control of P*_xylA_*. Again, the phenotype of the *uppP*-depletion strain (TMB3740) is more severe than that of the *bcrC*-depletion (TMB3739), in line with the morphological defects observed above (**Figure 3**). The additional deletion of *dgkA* or *yodM* had no effect on the observed MIC in any of the mutants tested, again indicating a very minor contribution of these two gene products. Removing the main bacitracin resistance determinant, *bceAB*, lead to overall lower basal MICs, but had no additional influence on the behavior described above (Supplementary Figure S7).

## Discussion

### Together, *bcrC* and *uppP* Encode the Essential UPP Phosphatase Function of *B. subtilis*

In our study, we demonstrated that *uppP* and *bcrC* constitute a synthetic lethal gene pair – a result that is in perfect agreement with an independent study performed in parallel using CRISPR-dCas9 knock-downs to study the effect of UPP phosphatase levels in *B. subtilis* (Zhao et al., 2016). These observations thereby correct two previous studies, which independently reported the successful construction of a *uppP/bcrC* double deletion mutant that showed the same phenotype as a single *bcrC* deletion mutant (Bernard et al., 2005; Inaoka and Ochi, 2012). Both groups used deletion constructs based on the vector pMUTIN, which disrupted either *uppP* or *bcrC* by integrating via single homologous recombination (Vagner et al., 1998; Kobayashi et al., 2003; Bernard et al., 2005; Inaoka and Ochi, 2012). Based on their data, it must be postulated that these deletion constructs generated a gene fragment up- or downstream the integration site, which was still large enough to maintain (residual) UPP phosphatase activity. In contrast, both recent studies only used complete allelic replacement mutants based on double homologous recombination of a resistance cassette (this study and Zhao et al., 2016).

One important difference between the latter two studies is the occurrence of suppressor mutants in zone-of-inhibition assays, as reported by Zhao et al. (2016). In the timescale of our experiments, we did not observe such suppressors, most likely due to the low but detectable leakiness of the xylose-dependent expression system. While the xylose system is tight enough to allow observing morphological defects and envelope stress in our study, it is not as tight as the CRISPR-dCas9 system that was used in the parallel study (Zhao et al., 2016). This approach obviously generated a much higher selective pressure that ultimately lead to the occurence of suppressors. According to our results, the basal expression of *bcrC* driven by P*_xylA_* is still sufficient for growth at normal doubling times. This is in agreement with the parallel study, which initially failed to generate *uppP* or *bcrC* depletion strains due to the high basal activity of P*_spac(hy)_* which was used for complementation: The strains still grew in the absence of IPTG, despite the knock-out of the native *uppP* and *bcrC* genes (Zhao et al., 2016).

In contrast to BcrC and UppP, the third UPP phosphatase, YodM, did not provide a measurable contribution to the cellular UP pool. While the Zhao et al. (2016) study demonstrated that the gene product of *yodM* indeed has UPP phosphatase activity, this was only sufficient to support growth if expression was artificially improved, in line with our own observations. This is not surprising, considering the expression profiles from a comprehensive transcriptome study, which demonstrates a lack of *yodM* expression, but instead a strong counter-transcription (Supplementary Figurexs S1). Together, the data provided in this study and the recent report from the Helmann group unequivocally demonstrates that the UPP phosphatase activity in *B. subtilis* is primarily – if not exclusively – provided by BcrC and UppP. While both can functionally complement each other, our study indicates that the two phosphatases have slightly different functions in wild type cells.

### BcrC Is More Relevant during Vegetative Growth, While UppP Is Important for Efficient Sporulation

**Table 1** summarizes the main findings of our study with regard to bacitracin sensitivity, P*_bcrC_* activity, cell morphology and growth rates. While our data demonstrates that either phosphatase is sufficient to support growth, the respective mutants do show significant differences in their overall behavior. The Δ*bcrC* single mutant had a decreased MIC for bacitracin and an elevated P*_bcrC_* activity, in contrast to the Δ*uppP* strain. If the UPP phosphatase levels are further reduced or if the cultures are additionally challenged with bacitracin, the phenotypes are overall less severe if BcrC is complemented compared to UppP under similar conditions. Hence, the *uppP* depletion strain (that completely lacks BcrC) shows a severe growth defect and strong CESR in the absence of xylose. While an elevated P*_bcrC_* activity is also measured for the *bcrC* depletion strain, this effect is rather weak in the absence of bacitracin and only a mild growth defect is observed. Our data therefore not only supports previous findings that BcrC is the major UPP phosphatase during vegetative growth in *B. subtilis* (Bernard et al., 2005; Inaoka and Ochi, 2012), but also demonstrates that an ectopically complementing copy of *bcrC* is more efficient in providing the UPP phosphatase activity than a similar construct for *uppP*.

**Table 1:** Summary of phenotypes associated with UPP phosphatase mutants.

Strains		Phenotypes^1^			
Genotype (condition)	Phosphatase presence	Morphology (bulging)	CESR (P*_bcrC_*)^3^	Sensitivity (bacitracin)	Sporulation defect^5^
Wild type	BcrC, UppP	-	-	-	-
Δ*uppP*	BcrC	-	-	-	+
Δ*bcrC* Δ*uppP* P*_xylA_*-*bcrC* (xyl+)	BcrC	-	-	o	+
Δ*bcrC* Δ*uppP* P*_xylA_*-*bcrC* (xyl-)	(BcrC)^2^	+	+	+	++
Δ*bcrC*	UppP	-	oo	o	-
Δ*bcrC* Δ*uppP* P*_xylA_*-*uppP* (xyl+)	UppP	-	-	o	oo
Δ*bcrC* Δ*uppP* P*_xylA_*-*uppP* (xyl-)	(UppP)^2^	++	++^4^	++	++

^1^*Severity of phenotype (for details see corresponding results section): -, none; o, weak; oo, medium; +, strong; ++, very strong. ^2^Residual amounts of these phosphatases are based on the slight leakiness of P_*xylA*_. ^3^CESR expressed as increased P_*bcrC*_ activity in stationary phase. ^4^In this strain, the P_*bcrC*_ activity is even increased in exponential growth phase. ^5^Decreased amount of mature spores after 24 and 48 h growth in MCSEC medium*.

In contrast, UppP seems to play the more prominent role with regard to sporulation. At strongly reduced levels of UppP, BcrC can support but never fully compensate the function of UppP. Its role in the formation of mature spores was recently also observed in a screen for sporulation mutants (Meeske et al., 2016).

When triggering a CESR, as monitored by an increased σ^M^-dependent P*_bcrC_* activity (this study, Cao and Helmann, 2002; Radeck et al., 2016b), we recently observed that a deletion of *bcrC* further increases the CESR (Radeck et al., 2016a,b). Here, we could demonstrate that this effect holds true for UPP phosphatases in general: While a *uppP* deletion alone does not trigger the CESR, very low levels of UPP phosphatase activity (especially in the *uppP* depletion strain) cause a stronger CESR than the *bcrC* single mutant (**Figure 5**). This finding perfectly fits to the working model that low levels of UP (or downstream effects thereof) are the stimulus for σ^M^ activation, rather than the protein levels of BcrC (Lee and Helmann, 2013; Zhao et al., 2016). It is also supported by the finding that P*_bcrC_* is induced in presence of bacitracin, which blocks the dephosphorylation to UP by binding to UPP (Cao and Helmann, 2002).

### Outlook and Open Questions

Our study clearly demonstrates the essential role of UPP phosphatases for the lipid II cycle, in perfect agreement with results from an independent study performed in parallel (Zhao et al., 2016). In addition, we could demonstrate that these phosphatases also provide a direct link in connecting cell envelope homeostasis with CESR. Nevertheless, some questions are still open and need to be addressed in subsequent studies.

Quite surprisingly, P*_bcrC_* activity is unchanged in the *uppP* depletion strain treated with xylose, while the native *uppP* copy present in Δ*bcrC* is not sufficient to prevent CESR. This phenomenon could not be observed with regard to the bacitracin sensitivity and provokes the question if and how *uppP* is regulated during growth and CES. For further investigations, protein and/or activity levels of UPP phosphatases and the abundance of UPP and UP in challenged and non-challenged cells will help to better understand the stoichiometry of UPP dephosphorylation, a crucial step of the lipid II cycle. The lack of P*_liaI_* induction in UPP phosphatase-depleted strains – that was also observed by Zhao et al. (2016) – seems to be even more puzzling. Such a bottleneck in cell wall biosynthesis should result in perturbations affecting envelope integrity and hence activate P*_liaI_*. While we do not have an explanation for this behavior, it is reminiscent to the lack of response to bacitracin of P*_liaI_* in cell wall-less L-forms of *B. subtilis* (Wolf et al., 2012). Here, the lack of a P*_liaI_* response in the presence of bacitracin and other cell wall antibiotics was attributed to the absence of an intact cell wall biosynthesis machinery. If this would be true, it is tempting to postulate that the severe depletion of UPP phosphatases – as done in the present study – somehow also affects the integrity of the cell wall biosynthesis machinery, thereby removing the (still unknown) source of P*_liaI_* induction in the presence of peptide antibiotics that interfere with the lipid II cycle. But such a farfetched (and far reaching) speculation will require follow-up experiments.

Another aspect that needs to be taken into account is the substantial contribution of UPP *de novo* synthesis to the lipid II cycle: Reducing the UppS protein levels by 50% significantly altered cell wall antibiotic sensitivities (Lee and Helmann, 2013). But so far, very little is known about the stoichiometry between UPP recycling and *de novo* synthesis, which are both essential and depend on UPP phosphatases.

Two reactions are known to generate UP independent of UPP phosphatases: (i) recycling from WTA-shuttling, which depends on UP and is therefore not self-sustaining (Brown et al., 2013), and (ii) phosphorylation of undecaprenol, e.g., via DgkA (Jerga et al., 2007; Van Horn and Sanders, 2012). The cellular abundance and dynamics of undecaprenol has so far not been studied for *B. subtilis*, but data from other species indicates that this molecule is present in the membrane of Gram-positive bacteria and absent in Gram-negative bacteria (Higashi et al., 1970; Barreteau et al., 2009). In *Staphylococcus aureus*, a UP phosphatase activity was detected, but could not be assigned to a certain protein (Willoughby et al., 1972). Future studies – particularly for *B. subtilis* – will hopefully address the source of undecaprenol and its role as a possible resource for the lipid II cycle.

The localization and cellular dynamics of UPP phosphatases throughout the growth cycle and into sporulation might provide further insights into their activity pattern and hence their cellular roles. Such studies would also allow studying their proximity to active cell wall biosynthesis clusters (peptidoglycan and WTA), which could be a relevant proxy for efficient carrier supply (Kawai et al., 2011; Typas et al., 2012). Unfortunately, our initial attempts to generate functional translational GFP-fusions to the N- or C-terminus of UppP or BcrC were not successful. Some fusion constructs did not provide (sufficient) UPP phosphatase activity to complement the synthetic lethal gene pair in a *uppP* and *bcrC* deletion background. And those constructs that maintained the phosphatase activity lacked a fluorescent signal, potentially due to the fluorophore localizing to the extracellular side of the membrane (data not shown). Future studies, that employ protein linkers or fluorophors that mature in the periplasm (such as mCherry or superfolder GFP, Dammeyer and Tinnefeld, 2012) will hopefully circumvent these obstacles.

The data provided by our and other recent studies (Meeske et al., 2016; Zhao et al., 2016) are an important first step in gaining a mechanistic understanding on UPP phosphatases. But despite the insights gained during these studies, there is still a lot to be learned about the dynamics of the UP pool and how the functions that make and break this essential intermediate of cell envelope biosynthesis contribute to cell growth, differentiation and cellular stress responses.

## Experimental Procedures

### Bacterial Strains and Growth Conditions

*Escherichia coli* strains were routinely grown in lysogeny broth (LB) and *B. subtilis* in *M*OPS-based *c*hemically defined medium with *s*uccinate and glutamate (MCSE) (Radeck et al., 2013), supplemented with casamino acids (1%, CAA) and L-threonine (50 μg ml^-1^) (MCSEC) at 37°C with agitation (220 rpm). Addition of CAA was necessary to prevent background activity of P*_hom_*, which is located upstream of the integration site of the *uppP* and *bcrC* complementation constructs (Radeck et al., 2013). Transformations of *B. subtilis* were carried out as described previously (Harwood and Cutting, 1990). All *B. subtilis* strains used in this study are derivatives of the laboratory wild type strain W168 and are listed in Supplementary Table S1. All allelic replacements are shown as gene deletions in the main text and figure captions for better readability. Selective media for *E. coli* contained ampicillin (100 μg ml^-1^) or chloramphenicol (35 μg ml^-1^). Selective media for *B. subtilis* contained chloramphenicol (5 μg ml^-1^), kanamycin (10 μg ml^-1^), spectinomycin (200 μg ml^-1^), tetracycline (12.5 μg ml^-1^) and/or a combination of erythromycin (1 μg ml^-1^) and lincomycin (25 μg ml^-1^) for macrolide-lincosamide-streptogramin B (MLS) resistance. Solid media additionally contained 1.5% (w/v) agar. For complementation studies, full induction of the promoter P*_xylA_* was achieved by adding xylose to a final concentration of 0.2% (w/v). Overnight cultures contained xylose per default to ensure normal growth of depletion strains.

### DNA Manipulation

Plasmids were generated by using standard cloning techniques (Sambrook and Russell, 2001) with enzymes and buffers from New England Biolabs^®^ (NEB) according to the manufacturer’s protocols. Phusion^®^ polymerase was used for polymerase chain reaction (PCR) amplification for cloning purposes, otherwise OneTaq^®^ was used. PCR purification was performed with *HiYield PCR Gel Extraction/PCR Clean-up Kit* (Süd-Laborbedarf Gauting, SLG^®^). For complementation studies, *uppP* or *bcrC* were placed under control of the xylose-inducible promoter P*_xylA_* inserted into the *thrC*-integration vector pBS4S. For measurements of promoter activity, promoter fragments spanning about 400 bp upstream of the Shine-Dalgarno sequence of the respective gene were cloned into pAH328, which carried the *luxABCDE* operon as an online luminescence reporter (Schmalisch et al., 2010). All plasmids generated during this study and a brief description of the construction are provided in Supplementary Table S2.

Allelic replacement mutations of *bcrC* and *uppP* were generated via long flanking homology PCRs, as described previously (Mascher et al., 2003). The integration of plasmids or DNA fragments into the *B. subtilis* genome via double recombination was verified with threonine auxotrophy (*thrC*) or colony PCR (*sacA, uppP, bcrC, yodM, dgkA*). All primer sequences are listed in Supplementary Table S3.

### Luciferase Assay

Luciferase activities of *B. subtilis* strains harboring promoter-*lux* fusions were assayed using a Synergy^TM^ NEOALPHAB multi-mode microplate reader from BioTek^®^ (Winooski, VT, United States). The reader was controlled using the software Gen5^TM^ (version 2.06). Hundred microliter culture volume were used per well in 96-well plates (black wall, clear bottom, clear lid, Greiner Bio-One). Incubation in the reader occurred at 37°C with linear agitation (567 cpm) and luminescence and OD_600_ were measured every 5 min. Strains were grown in MCSEC medium. Overnight cultures contained 0.2% xylose, to ensure protein production in complementation strains. (i) Day cultures (containing 0.2% xylose) were inoculated 1:5,000 from fresh overnight cultures, and strains were grown until exponential phase (OD_600_ = 0.1–0.4) (ii) Cells were harvested by centrifugation, washed twice in MCSEC, resuspended in MCSEC and the optical density was adjusted to OD_600_ = 0.025. (iii) 0.2% xylose was added if indicated and incubation in the reader occurred for 3 h. (iv) 30 μg ml^-1^ of bacitracin was added, if applicable, and the incubation and measurement continued for 17 h. Specific luminescence activity is given by the raw luminescence output (RLU) normalized by cell density (RLU/OD) (Radeck et al., 2013). For Supplementary Figure S5, cultures were handled as described, but the resuspended cultures (ii) were set to OD_600_ = 0.1 and 1:2, 1:4, 1:8, 1:16, and 1:100 dilutions thereof.

### Microscopy

Cell morphologies and sporulation frequencies were studied with an Olympus Microscope (AX70, 100x oil objective, camera XC10) and the accompanying software (Olympus cellSens Dimension 1.14). Phase contrast and GFP fluorescence channels (filter cube: U-MNIB, FF blue longpass, Ex. 470-490 nm, Em. > 515 nm) were used. The exposure time for the GFP-channel was 100 ms. Strains were grown as described above (see “Luciferase assay” (i)) and incubated for up to 48 h in flasks. The day cultures were only supplemented with 0.2% xylose if indicated in figure legends. Samples were taken at late exponential phase (OD_600_ ∼0.6–0.8, typically after 5–6 h), late/very late stationary phase (24 h/48 h post inoculation). Phase contrast pictures were adjusted in brightness and contrast to improve cell shape detection. All GFP-channel pictures were adjusted in brightness and contrast with the identical settings.

### Determination of Minimal Inhibitory Concentration

Bacitracin resistance in *B. subtilis* strains was determined using Etest^®^ strips on bacterial lawns (bioMérieux, Marcy l’Etoile, France), as described previously (Radeck et al., 2016b), with the following changes: (i) MCSEC medium was used instead of MH, (ii) overnight cultures contained 0.2% xylose, and (iii) day cultures, soft agar and agar plates contained 0.2% xylose, if applicable (see figure legends).

## Author Contributions

JR and TM conceptualized the study. NL and JR designed the experiments and generated the *B. subtilis* strains. JR performed MIC assays and coordinated the experimental work, NL performed all remaining experiments. JR and TM wrote the manuscript. All authors approved the manuscript and agree to be accountable for the content of the work.

## Conflict of Interest Statement

The authors declare that the research was conducted in the absence of any commercial or financial relationships that could be construed as a potential conflict of interest.

## References

[B1] Amiteye S. Kobayashi K. Imamura D. Hosoya S. Ogasawara N. Sato T. (2003). *Bacillus subtilis* diacylglycerol kinase (DgkA) enhances efficient sporulation. *J. Bacteriol.* 185 5306–5309. 10.1128/jb.185.17.5306-5309.2003 12923107 PMC180973

[B2] Anderson R. G. Hussey H. Baddiley J. (1972). The mechanism of wall synthesis in bacteria. The organization of enzymes and isoprenoid phosphates in the membrane. *Biochem. J.* 127 11–25. 10.1042/bj1270011 4627447 PMC1178555

[B3] Azevedo E. C. Rios E. M. Fukushima K. Campos-Takaki G. M. (1993). Bacitracin production by a new strain of *Bacillus subtilis*. Extraction, purification, and characterization. *Appl. Biochem. Biotechnol.* 42 1–7. 10.1007/BF02788897 8215347

[B4] Barreteau H. Magnet S. El Ghachi M. Touze T. Arthur M. Mengin-Lecreulx D. (2009). Quantitative high-performance liquid chromatography analysis of the pool levels of undecaprenyl phosphate and its derivatives in bacterial membranes. *J. Chromatogr. B Analyt. Technol. Biomed. Life Sci.* 877 213–220. 10.1016/j.jchromb.2008.12.010 19110475

[B5] Bernard R. El Ghachi M. Mengin-Lecreulx D. Chippaux M. Denizot F. (2005). BcrC from *Bacillus subtilis* acts as an undecaprenyl pyrophosphate phosphatase in bacitracin resistance. *J. Biol. Chem.* 280 28852–28857. 10.1074/jbc.M413750200 15946938

[B6] Botella E. Devine S. K. Hubner S. Salzberg L. I. Gale R. T. Brown E. D. (2014). PhoR autokinase activity is controlled by an intermediate in wall teichoic acid metabolism that is sensed by the intracellular PAS domain during the PhoPR-mediated phosphate limitation response of *Bacillus subtilis*. *Mol. Microbiol.* 94 1242–1259. 10.1111/mmi.12833 25315493

[B7] Breukink E. de Kruijff B. (2006). Lipid II as a target for antibiotics. *Nat. Rev. Drug Discov.* 5 321–332. 10.1038/nrd2004 16531990

[B8] Breukink E. van Heusden H. E. Vollmerhaus P. J. Swiezewska E. Brunner L. Walker S. (2003). Lipid II is an intrinsic component of the pore induced by nisin in bacterial membranes. *J. Biol. Chem.* 278 19898–19903. 10.1074/jbc.M301463200 12663672

[B9] Brown S. Santa Maria J. P. Jr. Walker S. (2013). Wall teichoic acids of gram-positive bacteria. *Annu. Rev. Microbiol.* 67 313–336. 10.1146/annurev-micro-092412-155620 24024634 PMC3883102

[B10] Cao M. Helmann J. D. (2002). Regulation of the *Bacillus subtilis bcrC* bacitracin resistance gene by two extracytoplasmic function σ factors. *J. Bacteriol.* 184 6123–6129. 10.1128/JB.184.22.6123-6129.2002 12399481 PMC151963

[B11] Coote J. G. (1972). Sporulation in *Bacillus subtilis*. Characterization of oligosporogenous mutants and comparison of their phenotypes with those of asporogenous mutants. *J. Gen. Microbiol.* 71 1–15. 10.1099/00221287-71-1-1 4625072

[B12] Dammeyer T. Tinnefeld P. (2012). Engineered fluorescent proteins illuminate the bacterial periplasm. *Comput. Struct. Biotechnol. J.* 3:e201210013. 10.5936/csbj.201210013 24688673 PMC3962181

[B13] Economou N. J. Cocklin S. Loll P. J. (2013). High-resolution crystal structure reveals molecular details of target recognition by bacitracin. *Proc. Natl. Acad. Sci. U.S.A.* 110 14207–14212. 10.1073/pnas.1308268110 23940351 PMC3761639

[B14] Egan A. J. Biboy J. van’t Veer I. Breukink E. Vollmer W. (2015). Activities and regulation of peptidoglycan synthases. *Philos. Trans. R. Soc. Lond. B Biol. Sci.* 370:20150031. 10.1098/rstb.2015.0031 26370943 PMC4632607

[B15] Eiamphungporn W. Helmann J. D. (2008). The *Bacillus subtilis* σM regulon and its contribution to cell envelope stress responses. *Mol. Microbiol.* 67 830–848. 10.1111/j.1365-2958.2007.06090.x 18179421 PMC3025603

[B16] El Ghachi M. Bouhss A. Blanot D. Mengin-Lecreulx D. (2004). The *bacA* gene of *Escherichia coli* encodes an undecaprenyl pyrophosphate phosphatase activity. *J. Biol. Chem.* 279 30106–30113. 10.1074/jbc.M401701200 15138271

[B17] El Ghachi M. Derbise A. Bouhss A. Mengin-Lecreulx D. (2005). Identification of multiple genes encoding membrane proteins with undecaprenyl pyrophosphate phosphatase (UppP) activity in *Escherichia coli*. *J. Biol. Chem.* 280 18689–18695. 10.1074/jbc.M412277200 15778224

[B18] Fan J. Jiang D. Zhao Y. Liu J. Zhang X. C. (2014). Crystal structure of lipid phosphatase *Escherichia coli* phosphatidylglycerophosphate phosphatase B. *Proc. Natl. Acad. Sci. U.S.A.* 111 7636–7640. 10.1073/pnas.1403097111 24821770 PMC4040569

[B19] Fenton A. K. El Mortaji L. Lau D. T. C. Rudner D. Z. Bernhardt T. G. (2016). CozE is a member of the MreCD complex that directs cell elongation in *Streptococcus pneumoniae*. *Nat Microbiol.* 2:16237. 10.1038/nmicrobiol.2016.237 27941863 PMC5486215

[B20] Guariglia-Oropeza V. Helmann J. D. (2011). *Bacillus subtilis* σV confers lysozyme resistance by activation of two cell wall modification pathways, peptidoglycan O-acetylation and D-alanylation of teichoic acids. *J. Bacteriol.* 193 6223–6232. 10.1128/JB.06023-11 21926231 PMC3209214

[B21] Guo R. T. Ko T. P. Chen A. P. Kuo C. J. Wang A. H. Liang P. H. (2005). Crystal structures of undecaprenyl pyrophosphate synthase in complex with magnesium, isopentenyl pyrophosphate, and farnesyl thiopyrophosphate: roles of the metal ion and conserved residues in catalysis. *J. Biol. Chem.* 280 20762–20774. 10.1074/jbc.M502121200 15788389

[B22] Harwood C. R. Cutting S. M. (1990). *Molecular Biological Methods for Bacillus.* Chichester: John Wiley & Sons.

[B23] Helmann J. D. (2016). *Bacillus subtilis* extracytoplasmic function (ECF) sigma factors and defense of the cell envelope. *Curr. Opin. Microbiol.* 30 122–132. 10.1016/j.mib.2016.02.002 26901131 PMC4821709

[B24] Higashi Y. Strominger J. L. Sweeley C. C. (1970). Biosynthesis of the peptidoglycan of bacterial cell walls. XXI. Isolation of free C55-isoprenoid alcohol and of lipid intermediates in peptidoglycan synthesis from *Staphylococcus aureus*. *J. Biol. Chem.* 245 3697–3702. 4248530

[B25] Inaoka T. Ochi K. (2012). Undecaprenyl pyrophosphate involvement in susceptibility of *Bacillus subtilis* to rare earth elements. *J. Bacteriol.* 194 5632–5637. 10.1128/JB.01147-12 22904278 PMC3458658

[B26] Ishihara H. Takoh M. Nishibayashi R. Sato A. (2002). Distribution and variation of bacitracin synthetase gene sequences in laboratory stock strains of *Bacillus licheniformis*. *Curr. Microbiol.* 45 18–23. 10.1007/s00284-001-0041-5 12029522

[B27] Jerga A. Lu Y. J. Schujman G. E. de Mendoza D. Rock C. O. (2007). Identification of a soluble diacylglycerol kinase required for lipoteichoic acid production in *Bacillus subtilis*. *J. Biol. Chem.* 282 21738–21745. 10.1074/jbc.M703536200 17535816

[B28] Jordan S. Hutchings M. I. Mascher T. (2008). Cell envelope stress response in Gram-positive bacteria. *FEMS Microbiol. Rev.* 32 107–146. 10.1111/j.1574-6976.2007.00091.x 18173394

[B29] Jordan S. Junker A. Helmann J. D. Mascher T. (2006). Regulation of LiaRS-dependent gene expression in *Bacillus subtilis*: identification of inhibitor proteins, regulator binding sites, and target genes of a conserved cell envelope stress-sensing two-component system. *J. Bacteriol.* 188 5153–5166. 10.1128/JB.00310-06 16816187 PMC1539951

[B30] Kawai Y. Marles-Wright J. Cleverley R. M. Emmins R. Ishikawa S. Kuwano M. (2011). A widespread family of bacterial cell wall assembly proteins. *EMBO J.* 30 4931–4941. 10.1038/emboj.2011.358 21964069 PMC3243631

[B31] Kelley L. A. Mezulis S. Yates C. M. Wass M. N. Sternberg M. J. (2015). The Phyre2 web portal for protein modeling, prediction and analysis. *Nat. Protoc.* 10 845–858. 10.1038/nprot.2015.053 25950237 PMC5298202

[B32] Kobayashi K. Ehrlich S. D. Albertini A. Amati G. Andersen K. K. Arnaud M. (2003). Essential *Bacillus subtilis* genes. *Proc. Natl. Acad. Sci. U.S.A.* 100 4678–4683. 10.1073/pnas.0730515100 12682299 PMC153615

[B33] Kobras C. M. Mascher T. Gebhard S. (2017). “Application of a *Bacillus subtilis* whole-cell biosensor (*PliaI-lux*) for the identification of cell wall active antibacterial compounds,” in *Antibiotics: Methods and Protocols*, ed. Sass P. (New York, NY: Springer), 121–131. 10.1007/978-1-4939-6634-9_7 27873249

[B34] Kramer N. E. Smid E. J. Kok J. De Kruijff B. Kuipers O. P. Breukink E. (2004). Resistance of Gram-positive bacteria to nisin is not determined by Lipid II levels. *FEMS Microbiol. Lett.* 239 157–161. 10.1016/j.femsle.2004.08.033 15451114

[B35] Laddomada F. Miyachiro M. M. Dessen A. (2016). Structural insights into protein-protein interactions involved in bacterial cell wall biogenesis. *Antibiotics* 5:E14. 10.3390/antibiotics5020014 27136593 PMC4929429

[B36] Lee Y. H. Helmann J. D. (2013). Reducing the level of undecaprenyl pyrophosphate synthase has complex effects on susceptibility to cell wall antibiotics. *Antimicrob. Agents Chemother.* 57 4267–4275. 10.1128/AAC.00794-13 23796923 PMC3754353

[B37] Manat G. Roure S. Auger R. Bouhss A. Barreteau H. Mengin-Lecreulx D. (2014). Deciphering the metabolism of undecaprenyl-phosphate: the bacterial cell-wall unit carrier at the membrane frontier. *Microb. Drug Resist.* 20 199–214. 10.1089/mdr.2014.0035 24799078 PMC4050452

[B38] Mascher T. Margulis N. G. Wang T. Ye R. W. Helmann J. D. (2003). Cell wall stress responses in *Bacillus subtilis*: the regulatory network of the bacitracin stimulon. *Mol. Microbiol.* 50 1591–1604. 10.1046/j.1365-2958.2003.03786.x 14651641

[B39] Mascher T. Zimmer S. L. Smith T. A. Helmann J. D. (2004). Antibiotic-inducible promoter regulated by the cell envelope stress-sensing two-component system LiaRS of *Bacillus subtilis*. *Antimicrob. Agents Chemother.* 48 2888–2896. 10.1128/AAC.48.8.2888-2896.2004 15273097 PMC478541

[B40] McCloskey M. A. Troy F. A. (1980). Paramagnetic isoprenoid carrier lipids. 2. Dispersion and dynamics in lipid membranes. *Biochemistry* 19 2061–2066. 10.1021/bi00551a009 6246919

[B41] Meeske A. J. Rodrigues C. D. Brady J. Lim H. C. Bernhardt T. G. Rudner D. Z. (2016). High-throughput genetic screens identify a large and diverse collection of new sporulation genes in *Bacillus subtilis*. *PLOS Biol.* 14:e1002341. 10.1371/journal.pbio.1002341 26735940 PMC4703394

[B42] Meeske A. J. Sham L. T. Kimsey H. Koo B. M. Gross C. A. Bernhardt T. G. (2015). MurJ and a novel lipid II flippase are required for cell wall biogenesis in *Bacillus subtilis*. *Proc. Natl. Acad. Sci. U.S.A.* 112 6437–6442. 10.1073/pnas.1504967112 25918422 PMC4443310

[B43] Minnig K. Barblan J. L. Kehl S. Moller S. B. Mauel C. (2003). In *Bacillus subtilis* W23, the duet σ^X^ σ^M^, two sigma factors of the extracytoplasmic function subfamily, are required for septum and wall synthesis under batch culture conditions. *Mol. Microbiol.* 49 1435–1447. 10.1046/j.1365-2958.2003.03652.x 12940998

[B44] Muchova K. Wilkinson A. J. Barak I. (2011). Changes of lipid domains in *Bacillus subtilis* cells with disrupted cell wall peptidoglycan. *FEMS Microbiol. Lett.* 325 92–98. 10.1111/j.1574-6968.2011.02417.x 22092867 PMC3433793

[B45] Nicolas P. Mäder U. Dervyn E. Rochat T. Leduc A. Pigeonneau N. (2012). Condition-dependent transcriptome reveals high-level regulatory architecture in *Bacillus subtilis*. *Science* 335 1103–1106. 10.1126/science.1206848 22383849

[B46] Ohki R. Giyanto Tateno K. Masuyama W. Moriya S. Kobayashi K., (2003). The BceRS two-component regulatory system induces expression of the bacitracin transporter, BceAB, in *Bacillus subtilis*. *Mol. Microbiol.* 49 1135–1144. 10.1046/j.1365-2958.2003.03653.x 12890034

[B47] Peters J. M. Colavin A. Shi H. Czarny T. L. Larson M. H. Wong S. (2016). A comprehensive, CRISPR-based functional analysis of essential genes in bacteria. *Cell* 165 1493–1506. 10.1016/j.cell.2016.05.003 27238023 PMC4894308

[B48] Piggot P. J. Coote J. G. (1976). Genetic aspects of bacterial endospore formation. *Bacteriol. Rev.* 40 908–962. 12736 10.1128/br.40.4.908-962.1976 PMC413989

[B49] Radeck J. Fritz G. Mascher T. (2016a). The cell envelope stress response of *Bacillus subtilis*: from static signaling devices to dynamic regulatory network. *Curr. Genet.* 63 79–90. 10.1007/s00294-016-0624-0 27344142

[B50] Radeck J. Gebhard S. Orchard P. S. Kirchner M. Bauer S. Mascher T. (2016b). Anatomy of the bacitracin resistance network in *Bacillus subtilis*. *Mol. Microbiol.* 100 607–620. 10.1111/mmi.13336 26815905

[B51] Radeck J. Kraft K. Bartels J. Cikovic T. Dürr F. Emenegger J. (2013). The *Bacillus* BioBrick Box: generation and evaluation of essential genetic building blocks for standardized work with *Bacillus subtilis*. *J. Biol. Eng.* 7:29. 10.1186/1754-1611-7-29 24295448 PMC4177231

[B52] Rietkötter E. Hoyer D. Mascher T. (2008). Bacitracin sensing in *Bacillus subtilis*. *Mol. Microbiol.* 68 768–785. 10.1111/j.1365-2958.2008.06194.x 18394148

[B53] Sambrook J. Russell D. W. (2001). *Molecular Cloning - A Laboratory Manual.* Cold Spring Harbor, NY: Cold Spring Harbor Laboratory Press.

[B54] Schmalisch M. Maiques E. Nikolov L. Camp A. H. Chevreux B. Muffler A. (2010). Small genes under sporulation control in the *Bacillus subtilis* genome. *J. Bacteriol.* 192 5402–5412. 10.1128/JB.00534-10 20709900 PMC2950494

[B55] Siewert G. Strominger J. L. (1967). Bacitracin: an inhibitor of the dephosphorylation of lipid pyrophosphate, an intermediate in the biosynthesis of the peptidoglycan of bacterial cell walls. *Proc. Natl. Acad. Sci. U.S.A.* 57 767–773. 10.1073/pnas.57.3.767 16591529 PMC335574

[B56] Storm D. R. Strominger J. L. (1973). Complex formation between bacitracin peptides and isoprenyl pyrophosphates. The specificity of lipid-peptide interactions. *J. Biol. Chem.* 248 3940–3945. 4350651

[B57] Thackray P. D. Moir A. (2003). SigM, an extracytoplasmic function sigma factor of *Bacillus subtilis*, is activated in response to cell wall antibiotics, ethanol, heat, acid, and superoxide stress. *J. Bacteriol.* 185 3491–3498. 10.1128/JB.185.12.3491-3498.2003 12775685 PMC156226

[B58] The UniProt Consortium (2017). UniProt: the universal protein knowledgebase. *Nucleic Acids Res.* 45 D158–D169. 10.1093/nar/gkw1099 27899622 PMC5210571

[B59] Tseng C. L. Shaw G. C. (2008). Genetic evidence for the actin homolog gene mreBH and the bacitracin resistance gene *bcrC* as targets of the alternative sigma factor SigI of *Bacillus subtilis*. *J. Bacteriol.* 190 1561–1567. 10.1128/JB.01497-07 18156261 PMC2258693

[B60] Typas A. Banzhaf M. Gross C. A. Vollmer W. (2012). From the regulation of peptidoglycan synthesis to bacterial growth and morphology. *Nat. Rev. Microbiol.* 10 123–136. 10.1038/nrmicro2677 22203377 PMC5433867

[B61] Vagner V. Dervyn E. Ehrlich S. D. (1998). A vector for systematic gene inactivation in *Bacillus subtilis*. *Microbiology* 144(Pt 11), 3097–3104. 10.1099/00221287-144-11-3097 9846745

[B62] Van Horn W. D. Sanders C. R. (2012). Prokaryotic diacylglycerol kinase and undecaprenol kinase. *Annu. Rev. Biophys.* 41 81–101. 10.1146/annurev-biophys-050511-102330 22224599 PMC3575517

[B63] Willoughby E. Higashi Y. Strominger J. L. (1972). Enzymatic dephosphorylation of C55-isoprenylphosphate. *J. Biol. Chem.* 247 5113–5115. 4341538

[B64] Wolf D. Dominguez-Cuevas P. Daniel R. A. Mascher T. (2012). Cell envelope stress response in cell wall-deficient L-forms of *Bacillus subtilis*. *Antimicrob. Agents Chemother.* 56 5907–5915. 10.1128/AAC.00770-12 22964256 PMC3486569

[B65] Wolf D. Kalamorz F. Wecke T. Juszczak A. Mäder U. Homuth G. (2010). In-depth profiling of the LiaR response of *Bacillus subtilis*. *J. Bacteriol.* 192 4680–4693. 10.1128/JB.00543-10 20639339 PMC2937411

[B66] Wolf D. Mascher T. (2016). The applied side of antimicrobial peptide-inducible promoters from Firmicutes bacteria: expression systems and whole-cell biosensors. *Appl. Microbiol. Biotechnol.* 100 4817–4829. 10.1007/s00253-016-7519-3 27102123

[B67] Zhao H. Sun Y. Peters J. M. Gross C. A. Garner E. C. Helmann J. D. (2016). Depletion of undecaprenyl pyrophosphate phosphatases disrupts cell envelope biogenesis in *Bacillus subtilis*. *J. Bacteriol.* 198 2925–2935. 10.1128/JB.00507-16 27528508 PMC5055597

[B68] Zweers J. C. Nicolas P. Wiegert T. van Dijl J. M. Denham E. L. (2012). Definition of the σW regulon of *Bacillus subtilis* in the absence of stress. *PLOS ONE* 7:e48471. 10.1371/journal.pone.0048471 23155385 PMC3498285

