# Supplementary material for: The Essential UPP Phosphatase Pair BcrC and UppP Connects Cell Wall Homeostasis during Growth and Sporulation with Cell Envelope Stress Response in Bacillus subtilis
Source: Front Microbiol. 2017 Dec 5;8:2403. doi: 10.3389/fmicb.2017.02403 (PMC5723303; doi:10.3389/fmicb.2017.02403)
Supplement: Supplementary file 1 [file Data_Sheet_1.pdf]

Supplemental Material

The Essential UPP Phosphatase Pair BcrC and UppP Connects Cell Wall Homeostasis during Growth and Sporulation with Cell Envelope Stress response in *Bacillus subtilis*

Jara Radeck[1, $], Nina Lautenschläger[1,$], Thorsten Mascher[1,*]
[$] these authors contributed equally to this work
[1] Thorsten Mascher, General Microbiology, Institut für Mikrobiologie, Technische Universität Dresden, Dresden, Germany

*** Correspondence:**
Thorsten Mascher
thorsten.mascher@tu-dresden.de

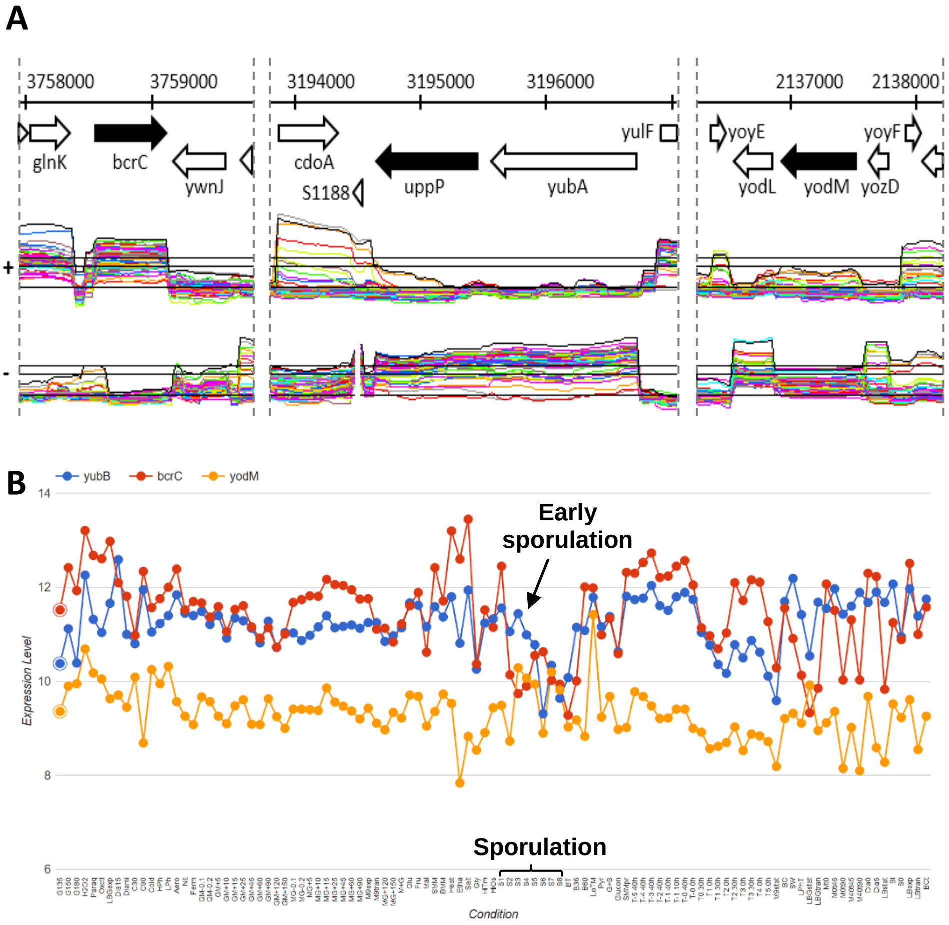

Figure S1. Expression of UPP-phosphatase genes. Figures modified from subtiwiki 2.0 (Nicolas et al., 2012; Michna et al., 2016). Details on the experimental conditions can be obtained there by clicking on the points of interest. **A**. Genomic context of UPP-phosphatase genes and respective mRNA levels (+ or – strand, respectively) across a variety of conditions. The scale indicates the genomic position. **B**. Comparison of expression profiles of *uppP (=yubB*, in blue*)*, *bcrC* (red) and *yodM* (yellow). The sporulation and early sporulation are indicated with a bracket and arrow, respectively.

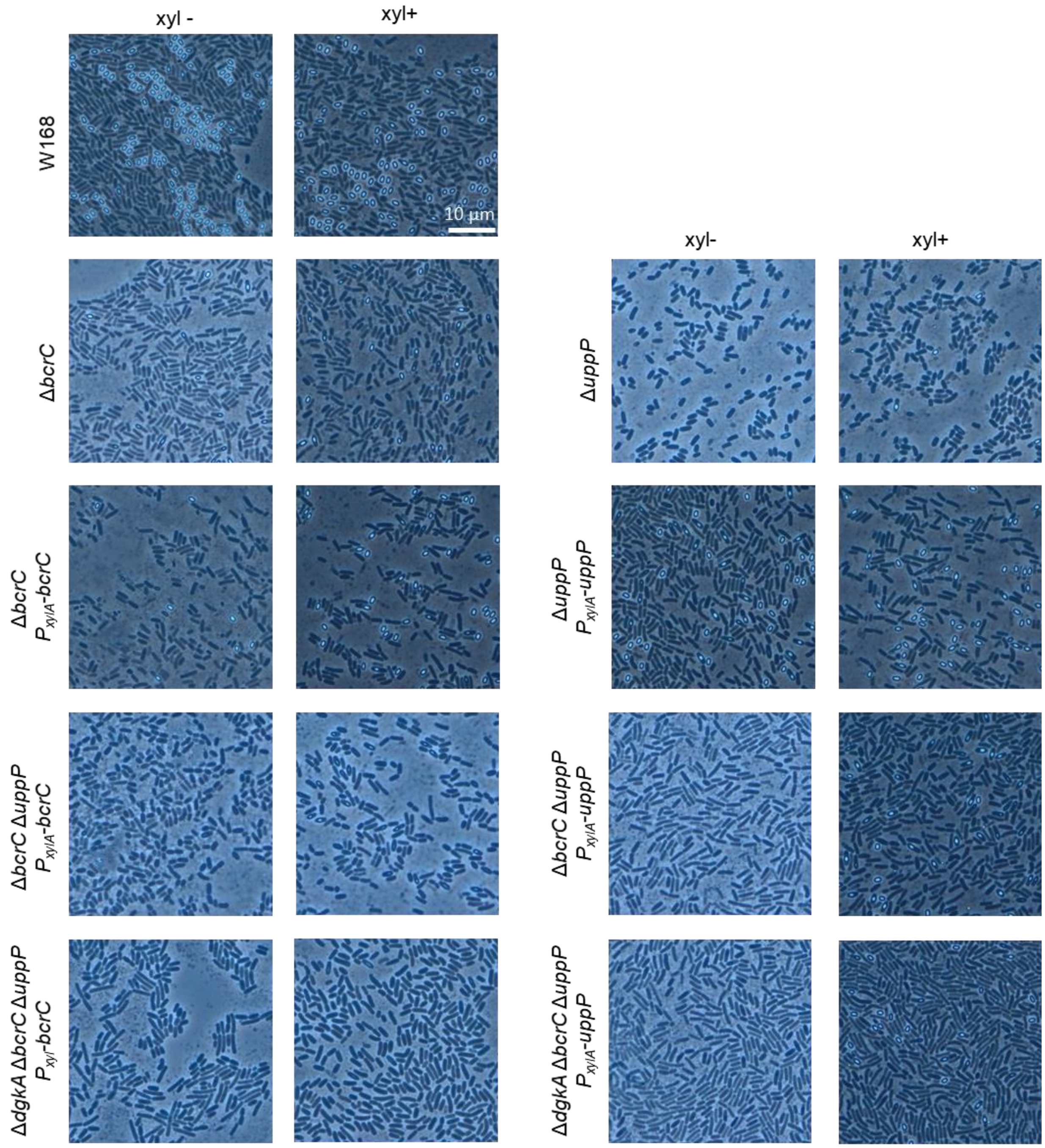

Figure S2. Sporulation efficiency of *bcrC* and *uppP* deletion and complementation mutants. Strains (W168, TMB297, TMB3694, TMB3739, TMB3957, TMB3408, TMB3695, TMB3740, and TMB3958) were grown as described in Fig. 2 and phase contrast microscope pictures were taken 24 h post-inoculation. The scale bar is 10 µm.

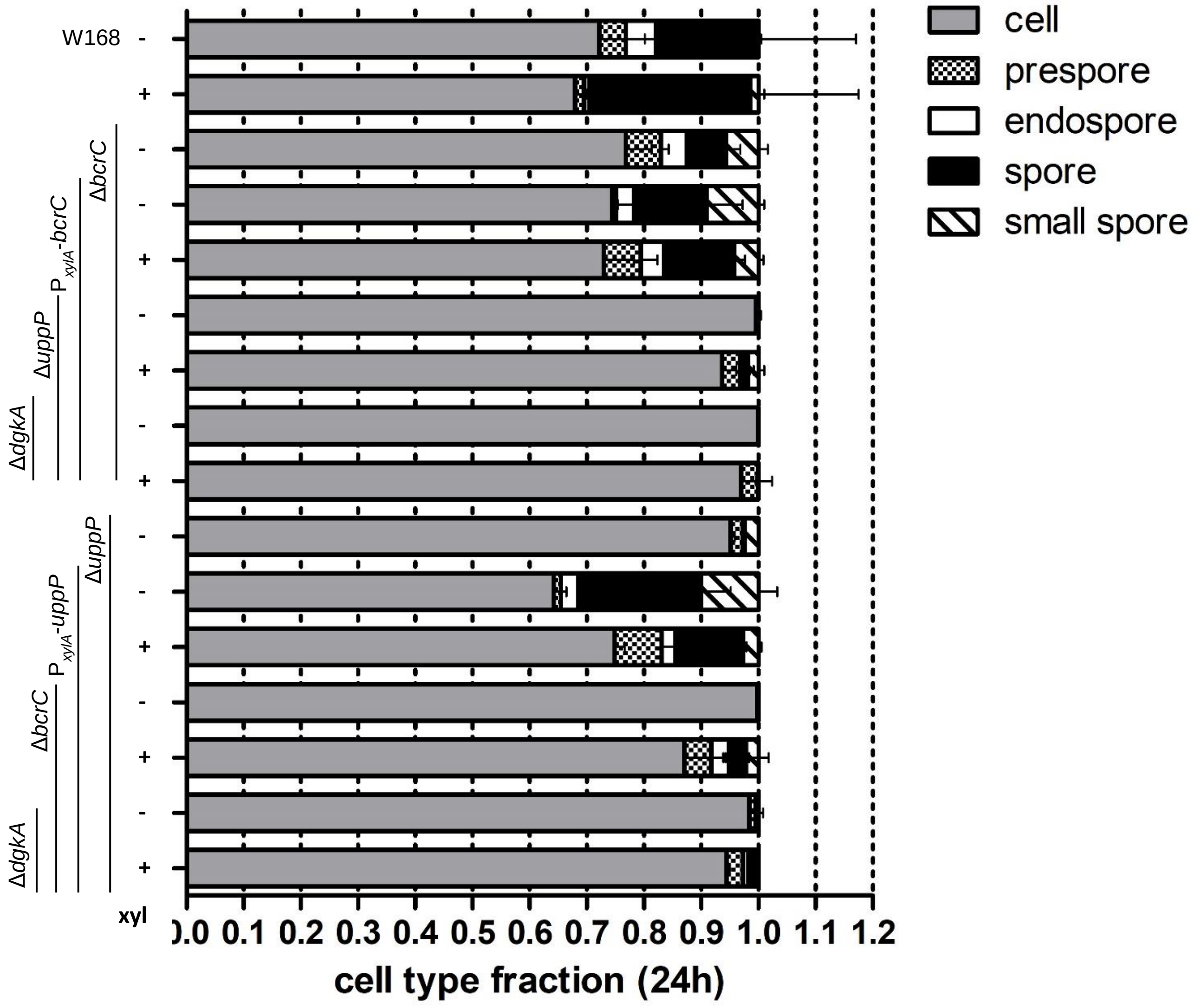

Figure S3. Sporulation efficiency of *bcrC* and *uppP* deletion and complementation mutants. Strains (W168, TMB297, TMB3694, TMB3739, TMB3957, TMB3408, TMB3695, TMB3740, and TMB3958) were grown as described in Fig. 2 and phase contrast microscopy pictures were taken 24 h post-inoculation. Legend: normal cells (grey), prespores without fully established phase-bright endospore (small checkered), completed endospores (white), free spores (black) and small free spores (striped). xyl+, 0.2% xylose supplemented in the day culture.

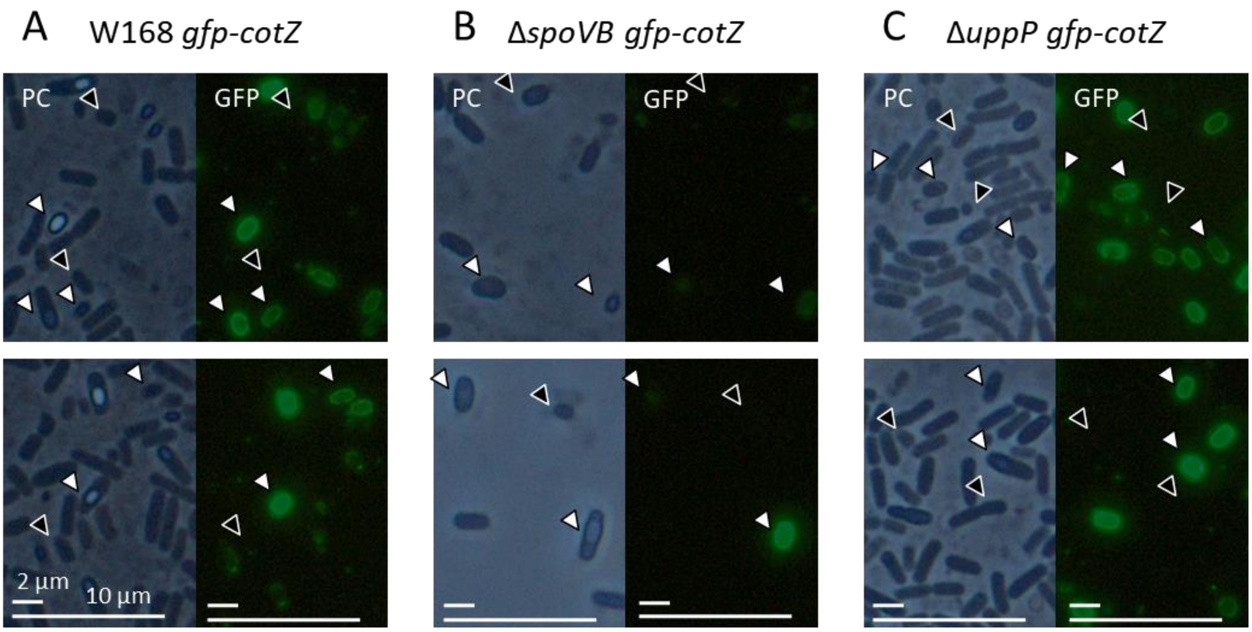

Figure S4. Detection of spores in *uppP* mutant via crust marker GFP-CotZ
Strains (TMB2112, TMB4150, and TMB4151) were grown as described in Fig. 3 and phase contrast as well as green fluorescence were documented 24 h post-inoculation. Prespores, endospores, free spores, phase-dark free spores, and small free spores can have fully developed spore crusts where GFP-CotZ is located (see white arrowheads with black border). However, not all small phase-dark particles can be classified as spores with this marker (see black arrowheads with white border, no GFP signal). **A**. **Wild type**. Please note that small spores only appear small in the phase contrast picture, but their crust has (almost) normal size as visualized in the GFP-channel. **B**. ***spoVB* mutant** (lipidII flippase, active during sporulation). Sporulation is strongly impaired. This strain was used as a control. **C**. ***uppP* mutant**. Hardly any developed phase-bright spores were formed. But prespores as well as some phase-dark spores (which could be mistaken for small cells) clearly show a spore crust. The scale bars are 2 or 10 µm, respectively.

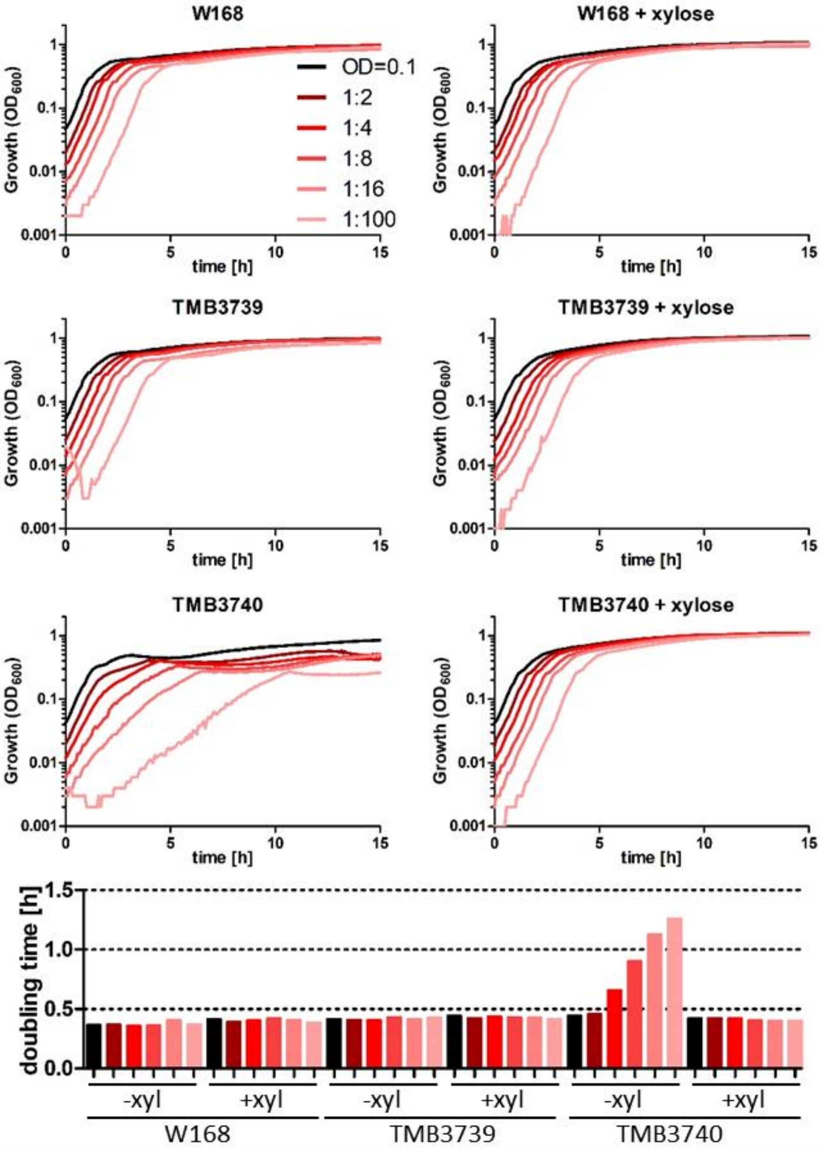

Figure S5. Growth of the wild type and *bcrC* and *uppP* complementation mutants. Strains were grown in MCSEC supplemented with 0.2% xylose at 37°C to OD_{600}=0.2-0.6, washed and resuspended to an optical density of OD_{600} = 0.1 in MCSEC (A, C, E), or MCSEC + 0.2% xylose (B, D, F). Cultures, as well as their 1:2, 1:4, 1:8, 1:16 and 1:100 dilutions were grown in 96-well plates in a microtiter plate reader at 37° where OD_{600} was measured every 5 minutes for 15 hours. **A-F.** Graphs show the OD_{600}-values as a measure for cell density, or the doubling time during exponential growth, with the color saturation decreasing with increasing dilutions. **A, B.** W168. **C, D.** TMB3739, Δ*bcrC* Δ*uppP* P_{xylA}-*bcrC*. **E, F.** TMB3740, Δ*bcrC* Δ*uppP* P_{xylA}-*uppP*. Data was obtained in biological triplicates, of which one representative sample is shown. **G.** The doubling times were calculated with Prism5, using the exponential growth equation and OD_{600}-values from 0.007 to 0.15 (TMB3740 –xyl) or 0.24 (all other strains), respectively.

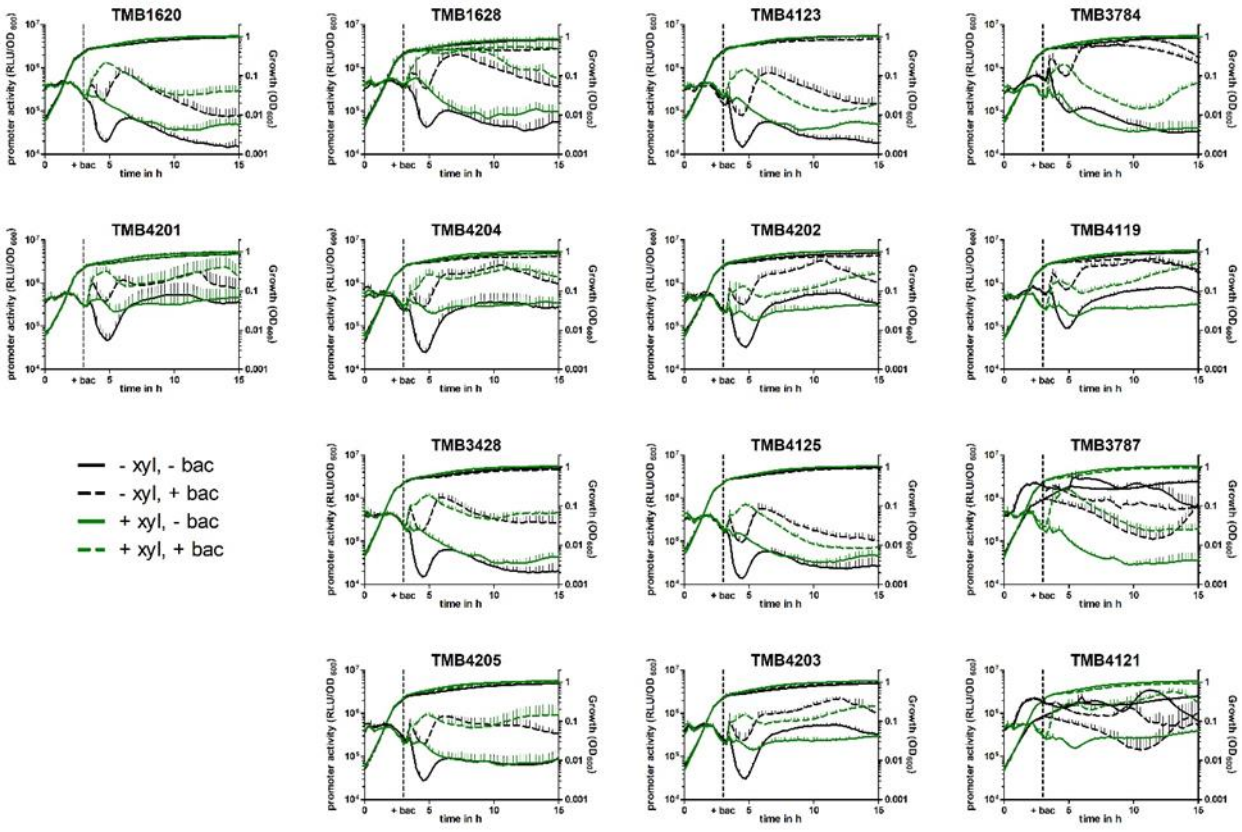

Figure S6. Growth and P$_{bcrC}$ promoter activities in the wild type and *bcrC* and *uppP* complementation mutants. See legend of Fig. 5. Black, without xylose; green, + 0.2% xylose; solid line, without bacitracin; dashed line, + 30 µg ml^{-1} bacitracin. Thin lines represent the standard deviation of three biological replicates.

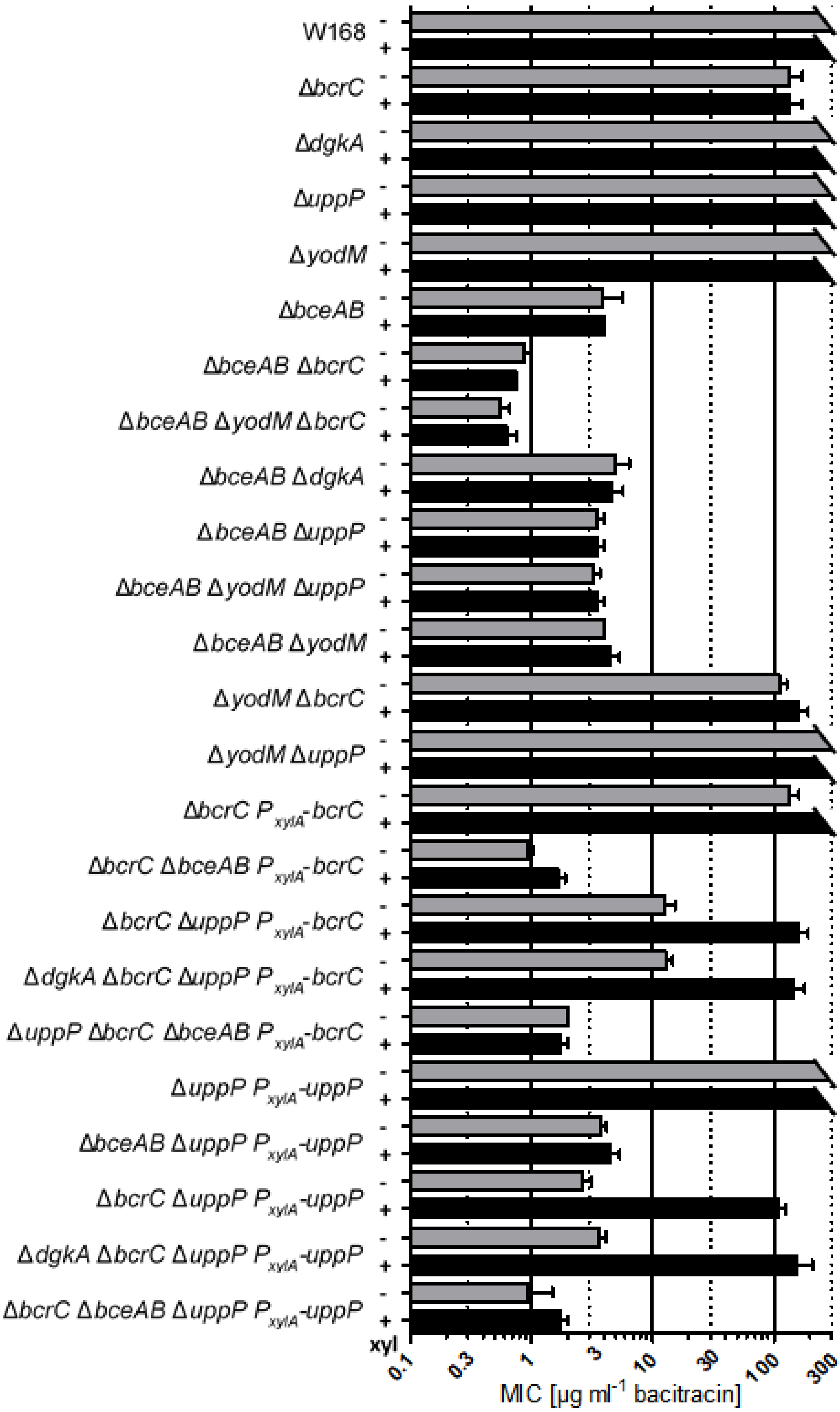

Figure S7. Minimal inhibitory bacitracin concentration of *bcrC* and *uppP* deletion and complementation mutants. Description, see Fig. 7.

Table S1: Bacterial strains used in this study

**Name**	**Description**[a]	**Source**
E. coli strains		
XL1-Blue	*recA1 endA1 gyrA96 thi-1 hsdR17 supE44 relA1 lac F'*::Tn*10* *proAB lacI*^{q} Δ(*lacZ*)M15]	Stratagene
NEB5α	*fhuA2 Δ(argF-lacZ)U169 phoA glnV44 Φ80 Δ(lacZ)M15 gyrA96 recA1 relA1 endA1 thi-1 hsdR17*	NEB
B. subtilis strains		
**W168**	wild type, *trpC2*	Laboratory stock
For luminescence analysis		
**TMB3688**	W168 *sacA*::pJRlux101 (P_{yubA}-*lux*)	This study
**TMB1620**	W168 *sacA*::pCHlux104 (P_{bcrC}-*lux*)	(Höfler et al., 2016)
**TMB1628**	W168 *bcrC*::*tet sacA*::pCHlux104 (P_{bcrC}-*lux*)	This study
**TMB4123**	W168 *bcrC*::*tet thrC*::pJR4S01 (P_{xylA}-*bcrC*) *sacA*::pCHlux104 (P_{bcrC}-*lux*)	This study
**TMB3784**	W168 *bcrC*::*tet uppP*::*MLS thrC*::pJR4S01 (P_{xylA}-*bcrC*) *sacA*::pCHlux104 (P_{bcrC}-*lux*)	This study
**TMB3428**	W168 *uppP*::*MLS sacA*::pCHlux104 (P_{bcrC}-*lux*)	This study
**TMB4125**	W168 *uppP*::*MLS thrC*::pJR4S02 (P_{xylA}-*uppP*) *sacA*::pCHlux104 (P_{bcrC}-*lux*)	This study
**TMB3787**	W168 *bcrC*::*tet uppP*::*MLS thrC*::pJR4S02 (P_{xylA}-*uppP*) *sacA*::pCHlux104 (P_{bcrC}-*lux*)	This study
**TMB4201**	W168 *dgkA::kan sacA*::pCHlux104 (P_{bcrC}-*lux*)	This study
**TMB4204**	W168 *dgkA::kan bcrC*::*tet sacA*::pCHlux104 (P_{bcrC}-*lux*)	This study
**TMB4202**	W168 *dgkA::kan bcrC*::*tet thrC*::pJR4S01 (P_{xylA}-*bcrC*) *sacA*::pCHlux104 (P_{bcrC}-*lux*)	This study
**TMB4119**	W168 *dgkA::kan bcrC*::*tet uppP*::*MLS thrC*::pJR4S01 (P_{xylA}-*bcrC*) *sacA*::pCHlux104 (P_{bcrC}-*lux*)	
**TMB4205**	W168 *dgkA::kan uppP*::*MLS sacA*::pCHlux104 (P_{bcrC}-*lux*)	This study
**TMB4203**	W168 *dgkA::kan uppP*::*MLS thrC*::pJR4S02 (P_{xylA}-*uppP*) *sacA*::pCHlux104 (P_{bcrC}-*lux*)	This study
**TMB4121**	W168 *dgkA::kan bcrC*::*tet uppP*::*MLS thrC*::pJR4S02 (P_{xylA}-*uppP*) *sacA*::pCHlux104 (P_{bcrC}-*lux*)	
Cell morphology, sporulation efficiency, and bacitracin MIC		
**TMB0297**	W168 *bcrC*::*tet*	(Rietkötter et al., 2008)
**TMB3694**	W168 *bcrC*::*tet thrC*::pJR4S01 (P_{xylA}-*bcrC*)	This study
**TMB3739**	W168 *bcrC*::*tet uppP*::*MLS thrC*::pJR4S01 (P_{xylA}-*bcrC*)	This study
**TMB3957**	W168 *dgkA::kan bcrC*::*tet uppP*::*MLS thrC*::pJR4S01 (P_{xylA}-*bcrC*)	This study
**TMB3408**	W168 *uppP*::*MLS*	This study
**TMB3695**	W168 *uppP*::*MLS thrC*::pJR4S02 (P_{xylA}-*uppP*)	This study
**TMB3740**	W168 *bcrC*::*tet uppP*::*MLS thrC*::pJR4S02 (P_{xylA}-*uppP*)	This study
**TMB3958**	W168 *dgkA::kan bcrC*::*tet uppP*::*MLS thrC*::pJR4S02 (P_{xylA}-*uppP*)	This study
GFP-CotZ		
**TMB4517**	W168 *amyE::*p1CSV-CotZ-N-GFP (P_{cotYZ}-*gfp-cotZ*)	(Julia Bartels, unpublished)
**TMB4150**	W168 *spoVB::MLS amyE::*p1CSV-CotZ-N-GFP (P_{cotY}-*gfp-cotZ*)	This study
**TMB4151**	W168 *uppP::MLS amyE::*p1CSV-CotZ-N-GFP (P_{cotY}-*gfp-cotZ*)	This study
Supplemental bacitracin MIC		
**TMB3923**	W168 *dgkA::kan*	This study
**TMB3568**	W168 *yodM::spec*	This study
**TMB0035**	W168 *bceAB::kan*	(Rietkötter et al., 2008)
**TMB0713**	W168 *bceAB:kan bcrC::tet*	(Radeck et al., 2016)
**TMB4104**	W168 *yodM::spec bcrC::tet bceAB::kan*	This study
**TMB4110**	W168 *dgkA::cat bceAB::kan*	This study
**TMB4102**	W168 *uppP::mls bceAB::kan*	This study
**TMB4105**	W168 *yodM::spec uppP::mls bceAB::kan*	This study
**TMB4103**	W168 *yodM::spec bceAB::kan*	This study
**TMB3716**	W168 *yodM::spec bcrC::tet*	This study
**TMB3738**	W168 *yodM::spec uppP::mls*	This study
**TMB4106**	W168 *bcrC*::*tet thrC*::pJR4S01 (P_{xylA}-*bcrC*) *bceAB::kan*	This study
**TMB4108**	W168 *bcrC*::tet *uppP::MLS thrC*::pJR4S01 (P_{xylA}-*bcrC*) *bceAB::kan*	This study
**TMB4107**	W168 *uppP::MLS thrC*::pJR4S02 (P_{xylA}-*uppP*) *bceAB::kan*	This study
**TMB4109**	W168 *bcrC::tet uppP::MLS thrC::*pJR4S02 (P_{xylA}-*uppP*) *bceAB::kan*	This study

Table S2. Vectors and plasmids used in this study

Name	Description	Resistance in *E. coli* / *B. subtilis*[a]	Primers and Enzymes used for cloning[b]	Source
**Vectors**				
pAH328	*sacA*'…'*sacA, luxABCDE, cat, bla*	Amp^r / cm^r		(Schmalisch et al., 2010)
pBS4S	*thrC*'…'*thrC*, *spc*, *bla*	Amp^r / spc^r		(Radeck et al., 2013)
pBS1C	*amyE'…'amyE, cat, bla*	Amp^r / cm^r		(Radeck et al., 2013)
**Plasmids**				
pCHlux104	pAH328-derivative, *sacA*::P_{bcrC}-*lux*, *cat*, *bla*	Amp^r / cm^r		(Höfler et al., 2016)
pJRlux101	pAH328-derivative, *sacA*::P_{yubA}-*lux*, *cat*, *bla*	Amp^r / cm^r	P_{yubA}: TM4738/TM5121; EcoRI, SalI	This study
pJR4S01	pBS4S-derivative, *thrC*::P_{xylA}-*bcrC*, *spc*, *bla*	Amp^r / spc^r	P_{xylA}:TM2968/ TM2969; EcoRI, SpeI.<br>*bcrC*: TM2731/ TM2732; XbaI ,PstI	This study
pJR4S02	pBS4S-derivative, *thrC*::P_{xylA}-*uppP, spc, bla*	Amp^r / spc^r	P_{xylA}:TM2968/ TM2969; EcoRI, SpeI.<br>*uppP*: 3 fragments (TM5122/ TM5125, TM5124/ TM5127, TM5126/ TM5123) were PCR-fused (TM5122/ TM5123)[c]; XbaI, PstI.	This study
p1CSV-CotZ-N-GFP	pBS1C-derivative, *amyE*::P_{cotYZ}-*gfp-cotZ, cat, bla*	Amp^r / cm^r		(Julia Bartels, unpublished)

[b] Amp^r, ampicillin resistance; cm^r, chloramphenicol resistance; spc^r, spectinomycin resistance.

[b] Genomic DNA of *B. subtilis* W168 was used as template for PCR. SpeI and XbaI generate compatible DNA overhangs. If two DNA-pieces were inserted, the vector was opened using the upstream restriction site of the promoter and the downstream restriction site of the gene, respectively.

[c] Two silent mutations with similar codon usage were introduced into *uppP* to allow cloning in BioBrick™ standard: P103: CCT→CCA, A195: GCA→GCG.

Table S3. Primers used in this study

Primer name	Description	Sequence (5'->3') [a]
**Primers used for cloning**		
TM4738	P_{yubA}-fwd	GATC**GAATTC**GCGGCCGCT**TCTAGA**GTTCGGGCTCGCTATGTATAC
TM5121	P_{yubA}-rev	TAA**GTCGAC**TCATACATAGTTTAATTAAATTGTACAC
TM2968	P_{xylA}-fwd	GATC**GAATTC**GCGGCCGCT**TCTAGA**GAAGGCCAAAAAACTGCTGCC
TM2969	P_{xylA}-rev	GATC**ACTAGT**ATTCGATAAGCTTGGGATCCC
TM2731	*bcrC*-fwd	GATC**GAATTC**GCGGCCGCT**TCTAGA**AAGGAGGTG**GCCGGC**TTGAACTACGAAATTTTTAAAGCAATC
TM2732	*bcrC*-rev	GATC**ACTAGT**ATTA**ACCGGT**GAAATTTTGATCGGTTGGTTTTTTC
TM5122	*uppP*-fwd	CCTA**GAATTC**GCGGCCGCT**TCTAGA**AAGGAGGTG**GCCGGC**ATGACTCTATGGGAATTGTTTG
TM5123	*uppP*-rev	GCCGGA**CTGCAG**CGGCCGCT**ACTAGT**ATTA**ACCGGT**TTACATCATGATCAAAAGTAAAATCAC
TM5124	*uppP*-PstImut1-fwd	CCGTCGGACTCGTGCC**a**GCAGCTGTTCTCGGCTTTTTG
TM5125	*uppP*-PstImut1-rev	CAAAAAGCCGAGAACAGCTGC**t**GGCACGAGTCCGACGG
TM5126	*uppP*-PstImut2-fwd	GATTAAACCACCGAGCTGC**g**GCCGACTTTACGTTTATTATGG
TM5127	*uppP*-PstImut2-rev	CCATAATAAACGTAAAGTCGGC**c**GCAGCTCGGTGGTTTAATC
**Primers used for LFH-PCRs**		
TM4749	*uppP*-up-fwd	GAGATTATCATTTCGATCGTCAC
TM4750	*uppP*-up-rev	CCTATCACCTCAAATGGTTCGCTGGTACTCTGTTAATCCTTCTACG
TM4751	*uppP*-do-fwd	CGAGCGCCTACGAGGAATTTGTATCGTTGCAATCTATCGAATTATTCTC
TM4752	*uppP*-do-rev	AATGGAACTGTATGAGTGTATCC
TM0139	MLS-fwd	CAGCGAACCATTTGAGGTGATAGGGATCCTTTAACTCTGGCAACCCTC
TM0140	MLS-rev	CGATACAAATTCCTCGTAGGCGCTCGGGCCGACTGCGCAAAAGACATAATCG
TM0057	MLS-check-fwd	CCTTAAAACATGCAGGAATTGACG
TM0148	MLS-check-rev	GTTTTGGTCGTAGAGCACACGG
TM5303	*dgkA*-up-fwd	CAAGAGTCGGCGCATATTATC
TM5304	*dgkA*-up-rev	CCTATCACCTCAAATGGTTCGCTGGAAATTCCGCTCCGTCCG
TM5305	*dgkA*-down-fwd	CGAGCGCCTACGAGGAATTTGTATCGCAGCCATTGAACATACGGTTG
TM5306	*dgkA*-down-rev	GGATAGAATTGCGGCCCTTC
TM0137	kan-fwd	CAGCGAACCATTTGAGGTGATAGG
TM0138	kan-rev	CGATACAAATTCCTCGTAGGCGCTCGG
TM0056	kan-check-fwd	CATCCGCAACTGTCCATACTCTG
TM0147	kan-check-rev	CTGCCTCCTCATCCTCTTCATCC
TM0135	cat-fwd	CAGCGAACCATTTGAGGTGATAGGCGGCAATAGTTACCCTTATTATCAAG
TM0136	cat-rev	CGATACAAATTCCTCGTAGGCGCTCGGCCAGCGTGGACCGGCGAGGCTAGTTACCC
TM0173	cat-check-fwd	CTAATGTCACTAACCTGCCC
TM0146	cat-check-rev	GTCTGCTTTCTTCATTAGAATCAATCC

[a] Recognition sites for endonuclease restriction enzymes are in bold. Introduced mutations are in bold lower case. The annealing part is underlined.

Supplemental References

Höfler, C., Heckmann, J., Fritsch, A., Popp, P., Gebhard, S., Fritz, G., et al. (2016). Cannibalism stress response in *Bacillus subtilis*. *Microbiology* 162(1), 164-176. doi: 10.1099/mic.0.000176.

Michna, R.H., Zhu, B., Mader, U., and Stulke, J. (2016). SubtiWiki 2.0--an integrated database for the model organism *Bacillus subtilis*. *Nucleic Acids Res* 44(D1), D654-662. doi: 10.1093/nar/gkv1006.

Nicolas, P., Mäder, U., Dervyn, E., Rochat, T., Leduc, A., Pigeonneau, N., et al. (2012). Condition-dependent transcriptome reveals high-level regulatory architecture in *Bacillus subtilis*. *Science* 335(6072), 1103-1106. doi: 10.1126/science.1206848.

Radeck, J., Gebhard, S., Orchard, P.S., Kirchner, M., Bauer, S., Mascher, T., et al. (2016). Anatomy of the bacitracin resistance network in *Bacillus subtilis*. *Mol Microbiol* 100(4), 607-620. doi: 10.1111/mmi.13336.

Radeck, J., Kraft, K., Bartels, J., Cikovic, T., Dürr, F., Emenegger, J., et al. (2013). The *Bacillus* BioBrick Box: generation and evaluation of essential genetic building blocks for standardized work with *Bacillus subtilis*. *J Biol Eng* 7(1), 29. doi: 10.1186/1754-1611-7-29.

Rietkötter, E., Hoyer, D., and Mascher, T. (2008). Bacitracin sensing in *Bacillus subtilis*. *Mol Microbiol* 68(3), 768-785. doi: 10.1111/j.1365-2958.2008.06194.x.

Schmalisch, M., Maiques, E., Nikolov, L., Camp, A.H., Chevreux, B., Muffler, A., et al. (2010). Small genes under sporulation control in the *Bacillus subtilis* genome. *J Bacteriol* 192(20), 5402-5412. doi: 10.1128/JB.00534-10.
